# Ribosomal L1 domain-containing protein 1 coordinates with HDM2 to negatively regulate p53 in human colorectal Cancer cells

**DOI:** 10.1186/s13046-021-02057-8

**Published:** 2021-08-06

**Authors:** Li Ding, Zhiping Zhang, Chenhong Zhao, Lei Chen, Zhiqiang Chen, Jie Zhang, Yaxian Liu, Yesen Nie, Yanzhi He, Kai Liao, Xinyue Zhang

**Affiliations:** 1 College of Bioscience and Biotechnology, Yangzhou University, Yangzhou, 225009 Jiangsu China; 2 Joint International Research Laboratory of Agriculture & Agri-Product Safety, The Ministry of Education of China, Yangzhou University, Yangzhou, 225009 Jiangsu China; 3 Key Laboratory of Prevention and Control of Biological Hazard Factors (Animal Origin) for Agrifood Safety and Quality, The Ministry of Agriculture of China, Yangzhou University (26116120), Yangzhou, 225009 Jiangsu China; 4 Jiangsu Co-innovation Center for Prevention and Control of Important Animal Infectious Diseases and Zoonoses, Yangzhou, 225009 Jiangsu China

**Keywords:** RSL1D1, Nucleolar protein, p53, HDM2, mRNA stability, Protein-RNA interaction, Ubiquitination, Protein-protein interaction, Cell proliferation, Cell survival

## Abstract

**Background:**

Ribosomal L1 domain-containing protein 1 (RSL1D1) is a nucleolar protein that is essential in cell proliferation. In the current opinion, RSL1D1 translocates to the nucleoplasm under nucleolar stress and inhibits the E3 ligase activity of HDM2 via direct interaction, thereby leading to stabilization of p53.

**Methods:**

Gene knockdown was achieved in HCT116^*p53+/+*^, HCT116^*p53−/−*^, and HCT-8 human colorectal cancer (CRC) cells by siRNA transfection. A lentiviral expression system was used to establish cell strains overexpressing genes of interest. The mRNA and protein levels in cells were evaluated by qRT-PCR and western blot analyses. Cell proliferation, cell cycle, and cell apoptosis were determined by MTT, PI staining, and Annexin V-FITC/PI double staining assays, respectively. The level of ubiquitinated p53 protein was assessed by IP. The protein-RNA interaction was investigated by RIP. The subcellular localization of proteins of interest was determined by IFA. Protein-protein interaction was investigated by GST-pulldown, BiFC, and co-IP assays. The therapeutic efficacy of RSL1D1 silencing on tumor growth was evaluated in HCT116 tumor-bearing nude mice.

**Results:**

RSL1D1 distributed throughout the nucleus in human CRC cells. Silencing of RSL1D1 gene induced cell cycle arrest at G1/S and cell apoptosis in a p53-dependent manner. RSL1D1 directly interacted with and recruited p53 to HDM2 to form a ternary RSL1D1/HDM2/p53 protein complex and thereby enhanced p53 ubiquitination and degradation, leading to a decrease in the protein level of p53. Destruction of the ternary complex increased the level of p53 protein. RSL1D1 also indirectly decreased the protein level of p53 by stabilizing HDM2 mRNA. Consequently, the negative regulation of p53 by RSL1D1 facilitated cell proliferation and survival and downregulation of RSL1D1 remarkably inhibited the growth of HCT116^*p53+/+*^ tumors in a nude mouse model.

**Conclusion:**

We report, for the first time, that RSL1D1 is a novel negative regulator of p53 in human CRC cells and more importantly, a potential molecular target for anticancer drug development.

**Supplementary Information:**

The online version contains supplementary material available at 10.1186/s13046-021-02057-8.

## Background

The nucleolus is a nuclear subcompartment for the synthesis and processing of nascent rRNAs and subsequent ribosome assembly in cells [1]. This intranuclear organelle is a highly complex and plurifunctional compartment [2, 3]. Upregulation of ribosome biogenesis contributes to neoplastic transformation by affecting the balance of protein translation and altering the synthesis of tumor-related proteins, such as c-Myc, HDM2, and p53 [4–7]. Many nucleolar and ribosomal proteins, including NPM [8], nucleostemin [9], RPL6 [10, 11], and RPL11 [12], play important roles in neoplasia. They regulate cell cycle progression [10], cell proliferation and apoptosis [9, 10], and the p53 signaling pathway [11–13].

Ribosomal L1 domain-containing protein 1 (RSL1D1) is a nucleolar protein encoded by cellular senescence-inhibited gene (CSIG) [14–16]. This protein contains a ribosomal L1 domain in the N-terminus and a lysine-rich domain in the C-terminus [17]. The expression of RSL1D1 is high in early passaged fibroblasts but declines during cellular senescence [18, 19]. Under normal conditions, RSL1D1 is mainly localized in the nucleolus. Upon various nucleolar stresses, such as treatment with low-dose actinomycin D (Act-D) and adriamycin or silencing of TIF-IA, RSL1D1 translocates to the nucleoplasm [20].

RSL1D1 regulates a wide range of cellular processes. It induces rRNA processing by destabilizing NOLC1 mRNA through direct interaction with its 5′-UTR [21]. It promotes UV-induced apoptosis by activating BAX [15]. Moreover, it regulates cellular replicative senescence and cell proliferation [16, 19, 22]. In a human 2BS fibroblast model, overexpression of RSL1D1 significantly promoted cell proliferation and delayed cellular replicative senescence, whereas downregulation of RSL1D1 expression reduced cell proliferation and accelerated cellular replicative senescence [19, 22].

RSL1D1 promotes cell proliferation by negatively regulating PTEN expression in HEK 293 and 2BS cells [19]. It interacts with the 5′-UTR of PTEN mRNA to suppress PTEN translation, in turn promoting cell proliferation [19]. RSL1D1 also prevents c-Myc ubiquitination via direct interaction to increase its level in HepG2 and SMMC7721 hepatocellular carcinoma cells, accordingly promoting cell proliferation [16]. PTEN and c-Myc are important regulators of tumorigenesis and metastasis and participate in the p53 signaling pathway [23–25]. These data indicate a strong association between RSL1D1 and p53. Recently, Xie, et al has reported that RSL1D1 translocates from the nucleolus to the nucleoplasm in response to nucleolar stress and interacts directly with the C-terminal RING finger domain of HDM2, a primary negative regulator of p53, to inhibit its E3 ubiquitin ligase activity. This in turn stabilizes p53 and arrests cell cycle progression [20]. Here, we report a reverse function of RSL1D1 in the regulation of p53 in HCT116 and HCT-8 colorectal cancer (CRC) cells, which might result from the distribution of RSL1D1 in the entire nucleus of CRC cells under normal conditions. In our model, RSL1D1 acts as an oncoprotein that negatively regulate p53 activity by stabilizing HDM2 mRNA and recruiting p53 to HDM2 via direct interaction for ubiquitination and degradation, thereby leading to cancer cell proliferation and survival.

## Methods

### Mice

All animal experiments were approved by the Institutional Animal Care and Use Committee of Yangzhou University and complied with the guidelines of the Jiangsu Laboratory Animal Welfare and Ethical Committee of Jiangsu Administrative Committee of Laboratory Animals. To evaluate the efficacy of siRNA against RSL1D1 (siRSL1D1) in antitumor therapy in female BALB/c-*Foxn1*^*nu*^/Nju nude mice, 2.5 × 10^6^ HCT116 cells were injected into the upper right axillary fossa of 4- to 6-week-old mice with a body weight of 18–22 g. When tumors grew to 0.3–0.4 cm in diameter, the mice were randomly divided into two groups (*n* = 5) and treated *diebus tertius* with siNC-PEI or siRSL1D1-PEI mixtures via percutaneous intratumor injection. The antitumor effect was evaluated by determining the mean tumor volume of mice in each group. The mice were sacrificed after a 15-day treatment course. All tumors were photographed and divided into two parts. One part was subjected to H&E staining analysis and the other part was used for western blot analysis to determine the protein level of RSL1D1 protein in the tumors. β-actin was used as a loading control.

### Cell culture

HCT116^*p53+/+*^ and HCT-8 cells were kindly provided by the Cell Bank of the Chinese Academy of Sciences. HCT116^*p53−/−*^ cells were a gift from Dr. Bert Vogelstein of Johns Hopkins University. Lenti-X™ 293 T cells were purchased from Takara Biomedical Technology Co., Ltd. (Beijing, China). HCT116^*p53+/+*^ and HCT116^*p53−/−*^ cells were cultured in McCoy’s 5A medium, supplemented with 10% heat-inactivated fetal bovine serum, 2 mM L-glutamine, 100 U/mL penicillin and 100 mg/mL streptomycin. The Lenti-X™ 293 T and HCT-8 cells were cultured in DMEM medium with the same supplements. All cells were cultured at 37 °C in a humidified incubator with a 5% CO_2_ atmosphere.

### Lentivirus production and transduction

Lentiviral plasmid (pLVX-TetOne-Puro-EGFP, pLVX-TetOne-Puro-FLAG-RSL1D1, pLVX-TetOne-Puro-FLAG-RSL1D1-NT, pLVX-TetOne-Puro-FLAG-RSL1D1-CT, or pLenti6/V5-GW/p53), packaging plasmid pCMV-dR8.2, and envelop plasmid pCMV-VSV-G were co-transfected into Lenti-X™ 293 T cells (80 to 90% confluence) to produce lentiviruses using Lipofectamine™ 2000 (Invitrogen, Carlsbad, USA) according to the reagent manual. The lentiviruses were transduced into cells according to the Addgene pLKO.1 protocol (http://www.addgene.org/protocols/plko/). After transduction, cells were selected with 2 μg/mL puromycin (for pLVX-TetOne-Puro) or 10 μg/mL blasticidin S (for pLenti6/V5-GW/p53) for 5 days to establish stable cell strains, followed by treatment with 1 μg/mL puromycin or 10 μg/mL blasticidin S to maintain drug resistance. The stable cells were treated with 1 μg/mL doxycycline for 24 h (for RSL1D1-NT and RSL1D1-CT) or 72 h (for RSL1D1) to induce gene expression.

### siRNA-mediated knockdown of genes

siRNA molecules were designed to downregulate the expression of RSL1D1 (siRSL1D1), HDM2 (siHDM2), or FOXO3a (siFOXO3a). An unrelated siRNA sequence targeting *PLEKHB1* (GenBank accession no. XM_018572553.1) from *N. parkeri* was used as a negative control (siNC). The siRNA was transfected into cells using Lipofectamine™ 2000 according to the reagent manual and the medium was replaced 6 h later. Forty-eight hours post-transfection, the cells were harvested for qRT-PCR and western blot analyses to evaluate the knockdown efficiency. The siRNA sequences are listed in Supplementary Table S1.

### Total RNA extraction, cDNA synthesis, and qRT-PCR

Total RNA was isolated from cells using TRIzol according to the reagent manual. The RNA samples were reversely transcribed to cDNA using a HiFiScript cDNA Synthesis Kit (CoWin Biosciences). qRT-PCR was performed using EvaGreen 2× qPCR MasterMix (Applied Biological Materials Inc., Richmond, Canada). Reactions were run in a CFX96 Touch™ Real-time PCR system (Bio-Rad Laboratories, Hercules, USA) and data were analyzed using the built-in CFX Manager™ software. Data normalization was performed as previously described [26, 27]. The primer sequences are listed in Supplementary Table S1.

### Extraction of tissue, whole-cell, nuclear, and cytoplasmic proteins

For extraction of proteins from tissues or whole cells, tissue fragments or cells were resuspended in RIPA lysis buffer (Beyotime Biotechnology, Shanghai, China) containing 1× Protease Inhibitor Cocktail (Beyotime Biotechnology) and immediately homogenized (for tissue samples) or sonicated (for cell samples), followed by centrifugation at 12,000×g for 10 min at 4 °C. Nuclear and cytoplasmic proteins were prepared from the same amount of cells using the Nuclear and Cytoplasmic Protein Extraction Kit (Beyotime Biotechnology). Protein concentration was quantified using the Micro BCA Protein Assay Kit (CoWin Biosciences Co., Ltd., Beijing, China), followed by western blot analysis of the proteins of interest.

### Western blot analysis

Proteins were separated by SDS-PAGE and electrotransferred to a PVDF membrane. The membrane was blocked with 5% milk in PBST at room temperature for 1 h. Subsequently, the membrane was incubated with primary antibody at 4 °C overnight and then with HRP-labeled secondary antibody at room temperature for 1 h. Next, the membrane was incubated in UltraECL Chemiluminescence reagent (YuanPinHao Bio, Beijing, China) for 1 to 2 min. The specific bands were visualized using a Tanon-5200 chemiluminescence apparatus (Tanon Science & Technology Co. Ltd., Shanghai, China). The primary antibodies used in this study were against FLAG (mouse monoclonal M2, Cat# F1804, RRID# AB_262044, Sigma-Aldrich), 6× His (mouse monoclonal 2A11, Cat# CW0286, CoWin Biosciences), RSL1D1 (mouse monoclonal, homemade), p53 (mouse monoclonal DO-1, Cat# sc-126, RRID# AB_628082, Santa Cruz Biotechnology, Dallas, USA), HDM2 (rabbit monoclonal D1V2Z, Cat# 86934, RRID# AB_2784534, Cell Signaling Technology, Beverly, USA), p21 (rabbit monoclonal 12D1, Cat# 2974, RRID# AB_11217627, Cell Signaling Technology), PUMA (rabbit monoclonal D30C10, Cat# 12450, RRID# AB_2797920, Cell Signaling Technology), FOXO3a (rabbit monoclonal 75D8, Cat# AF609, Beyotime Biotechnology), HDM4 (rabbit polyclonal, Cat# ABP55215, Abbkine), β-actin (mouse monoclonal 3E8, Cat# CW0096M, RRID# AB_2736993, CoWin Biosciences), and HDAC1 (rabbit polyclonal, Cat# AH379, Beyotime Biotechnology).

### Proliferation of RSL1D1 knockdown HCT116 cells

siRSL1D1-transfected HCT116 cells were seeded into 96-well plates and incubated for 12 h to allow firm attachment to the bottom of the wells. The plates were then incubated in a 5% CO_2_ incubator at 37 °C for 0, 1, 2, and 3 days. Subsequently, MTT was added to each well. Four hours later, the culture medium was carefully removed and DMSO was added to each well to dissolve the formazan crystals. The OD values were determined using an Infinite M200 Pro 96-well microplate reader (Tecan Life Science, Männedorf, Switzerland) at 570 nm with a reference wavelength of 630 nm. The values were normalized against the absorbance of the wells seeded with siNC-transfected cells at day 0.

### Cell cycle and apoptosis analyses

PI staining analysis was performed to determine the distribution of each cell cycle phase. Briefly, the harvested cells were fixed and permeabilized with 70% ethanol at − 20 °C overnight. The cells were washed with 1× PBS and treated with 10 μg/mL of DNase-inactivated RNase A at room temperature for 1 h. Subsequently, the cells were incubated with 0.1 mg/mL of PI at room temperature in the dark for at least 10 min immediately prior to FACS analysis. Annexin V-FITC/PI staining analysis was performed to detect apoptosis according to the manufacturer’s manual. Briefly, the cells were harvested and washed twice with cold 1× PBS, followed by resuspension in 1× Annexin V binding buffer. Annexin V-FITC and PI were sequentially added into the suspension for staining. The stained cells were then loaded into a FACSCalibur flow cytometer (BD, Santa Clara, USA). Data were collected for cell cycle and apoptosis analyses using FlowJo v10 software (TreeStar, Ashland, USA).

### Immunoprecipitation (IP)

Cells were harvested and lysed in Cell Lysis Buffer for Western and IP (Beyotime Biotechnology) containing 1× Protease Inhibitor Cocktail (Beyotime Biotechnology). After ultrasonication and centrifugation at 12,000×g for 10 min at 4 °C, the protein concentration of each supernatant was determined. The supernatant was co-incubated with mouse anti-p53 monoclonal antibody (DO-1, Santa Cruz Biotechnology) at 4 °C for 4 h, followed by further incubation with Protein A + G beads (CoWin Biosciences) at 4 °C for 1 h. After washing four times with Cell Lysis Buffer, the beads were boiled in 1× SDS-PAGE sample loading buffer for western blot analysis with primary antibody against p53 (DO-1, Santa Cruz Biotechnology) and then with a secondary antibody against mouse IgG light chain (Abbkine, Wuhan, China). In addition, western blot analysis was performed to evaluate the levels of FLAG-RSL1D1, FLAG-RSL1D1-NT, FLAG-RSL1D1-CT, RSL1D1, p53, and HDM2 proteins in cell lysates (supernatants). β-actin was used as a loading control.

### Assessment of mRNA stability

Since high dose Act-D is reported to rapidly shut off mRNA transcription in the cultured cells and thereby be widely used to study the decay rates of remaining endogenous transcripts, we assessed the cellular mRNA stability of HDM2 following this classical approach [28]. After treatment with 4 μM Act-D, cells were harvested at different time points for qRT-PCR analysis of HDM2 mRNA levels. The extremely stable GAPDH mRNA was used as an internal control [28].

### RNA immunoprecipitation (RIP) assay

RIP assay was performed as previously described [29] with slight modifications. In detail, cells were incubated with 0.4% paraformaldehyde to cross-link RNA and protein at room temperature for 15 min and then with 0.2 M glycine for an additional 5 min to stop cross-linking. The cells were washed twice with PBS and lysed in RIP buffer containing 100 mM KCl, 5 mM MgCl_2_, 10 mM HEPES (pH 7.0), 0.5% NP40, 1 mM DTT, 1000 U/mL RNase Inhibitor (Beyotime Biotechnology), and 1× EDTA-free Protease Inhibitor Cocktail (Beyotime Biotechnology). After centrifugation at 12,000×g for 10 min at 4 °C, supernatants were co-incubated with anti-FLAG antibody (M2, Sigma Aldrich, St. Louis, USA) or mouse IgG at 4 °C overnight, followed by incubation with Protein A + G beads for 2 h. The beads were then washed four times with RIP buffer and treated with proteinase K to release RNA and protein components. TRIzol reagent was used to isolate RNA, followed by qRT-PCR analysis. RNA isolated directly from cell lysate was used as an input control.

### Immunofluorescence assay (IFA)

Cells were fixed in 4% paraformaldehyde at room temperature for 20 min, permeabilized in ice-cold 1× PBS containing 0.2% Triton X-100 for 10–15 min, and then blocked in 3% BSA in 1× PBS at room temperature for 1 h. The cells were incubated with mouse anti-RSL1D1 monoclonal antibody (homemade), rabbit anti-p53 monoclonal antibody (7F5, Cell Signaling Technology), or rabbit anti-HDM2 monoclonal antibody (D1V2Z, Cell Signaling Technology) at 4 °C overnight, followed by washing with 1× PBS three times. Subsequently, the cells were incubated with Cy3- or FITC-conjugated secondary antibody against mouse or rabbit IgG (Beyotime) at room temperature for 2 h. After washing with 1× PBS three times, the cells were stained with Hoechst 33258 and observed under the Leica TCS SP8 STED laser confocal microscope (Wetzlar, Germany).

### Expression and purification of recombinant proteins

The expression and purification of recombinant proteins were performed as we previously described [30]. In brief, a recombinant plasmid (pET-32a(+)-p53, pET-32a(+)-p53(1–92), pET-32a(+)-p53(1–292), pET-32a(+)-p53(1–363), pET-32a(+)-p53-DBD(92–292), pET-32a(+)-p53(293–393), pET-32a(+)-SUMO-HDM2, pGEX-6P-1-RSL1D1, pGEX-6P-1-RSL1D1-NT, or pGEX-6P-1-RSL1D1-CT) was transformed into competent *E. coli* BL21(DE3) for IPTG-induced expression of target genes. His- and GST-tagged recombinant proteins were purified using BeyoGold His-tag Purification Resin (Beyotime Biotechnology) and Glutathione Sepharose 4B (GE Healthcare, Chicago, USA), respectively. Protein purity was determined by SDS-PAGE analysis.

### GST-pulldown assay

The GST-pulldown assay was performed as previously described [31] with slight modifications. In brief, purified GST-tagged RSL1D1 protein was co-incubated with Glutathione Sepharose 4B beads at 4 °C for 1 h. Then, the beads were incubated with purified His-tagged p53 protein at 4 °C for 1 h, followed by washing five times with 1% Triton X-100 in PBS. The beads were boiled in SDS-PAGE sample loading buffer for western blot analysis.

### Bimolecular fluorescence complementation (BiFC) assay

The BiFC assay was performed to further explore the interaction between RSL1D1 and p53 in vivo according to a published protocol [32] with slight modifications. In brief, the coding regions of RSL1D1-FL, RSL1D1-NT and RSL1D1-CT were cloned into pBiFC-mCherryN159. The coding regions of p53-FL and p53-DBD were cloned into pBiFC-mCherryC160. HCT116^*p53+/+*^ cells were seeded into confocal dishes and co-transfected with recombinant plasmid pairs pBiFC-mCherryN159-RSL1D1-FL (or pBiFC-mCherryN159-RSL1D1-NT or pBiFC-mCherryN159-RSL1D1-CT) and pBiFC-mCherryC160-p53-FL (or pBiFC-mCherryC160-p53-DBD). Thirty-six hours post-transfection, the cells were incubated with Hoechst 33258 for nuclear staining and observed under the laser confocal microscope. Negative control cells were co-transfected with pBiFC-mCherryN159 and pBiFC-mCherryC160, whereas positive control cells were co-transfected with pBiFC-mCherryN159-SV40gp6 and pBiFC-mCherryC160-p53 because of the established p53-SV40gp6 interaction [33]. Relative fluorescent quantitative analysis was performed to assess the interaction between proteins using the software Image J (NIH, Bethesda, USA). To facilitate comparison, the mean level of fluorescence intensity derived from the p53-SV40gp6 interaction was set as 1.

A combination of BiFC and immunofluorescence assays was performed to investigate the intracellular colocalization of RSL1D1, p53, and HDM2. Briefly, after co-transfection with pBiFC-mCherryN159-RSL1D1-FL and pBiFC-mCherryC160-p53-FL, cells were subjected to fluorescent staining with anti-HDM2 antibody following the IFA protocol.

### Co-IP

Whole-cell proteins were extracted from lentivirus-transduced HCT116^*p53−/−*^ cells stably expressing V5-p53 or HCT116^*p53+/+*^ cells and incubated with anti-V5 (D3H8Q, Cell Signaling Technology) or anti-HDM2 antibody (D1V2Z, Cell Signaling Technology), respectively, at 4 °C overnight. Rabbit IgG was used as a negative control. The antibody-protein mixture was then incubated with Protein A + G beads at 4 °C for 2 h. After washing four times, the beads were boiled in 1× SDS-PAGE sample loading buffer for western blot analysis with a primary antibody against RSL1D1 (homemade), HDM2 (D1V2Z, Cell Signaling Technology), or p53 (DO-1, Santa Cruz Biotechnology). Cell lysate was used as an input control.

### Statistical analysis

All numerical data are presented as the mean ± SD. The significance of the difference between the mean values of the two groups was evaluated using Student’s t-test. Differences were considered statistically significant at *P* < 0.05 (*) and *P* < 0.01 (**).

## Results

### RSL1D1 is required for proliferation and survival of human colorectal Cancer cells

To investigate the function of RSL1D1 (GenBank accession no. NM_015659.3) in cancer cells, we first analyzed the expression of RSL1D1 in human cancer tissues and normal counterparts by interrogation of the Oncomine Cancer Microarray database (www.oncomine.org/). 49 out of all 73 independent datasets showed that RSL1D1 was significantly upregulated in cancer comparing with normal tissues (*P* < 0.001) (Supplementary Fig. S1). More importantly, RSL1D1 was upregulated in all 18 CRC datasets, suggesting that RSL1D1 might promote the proliferation and survival of CRC cells as an oncoprotein.

**Fig. 1:**
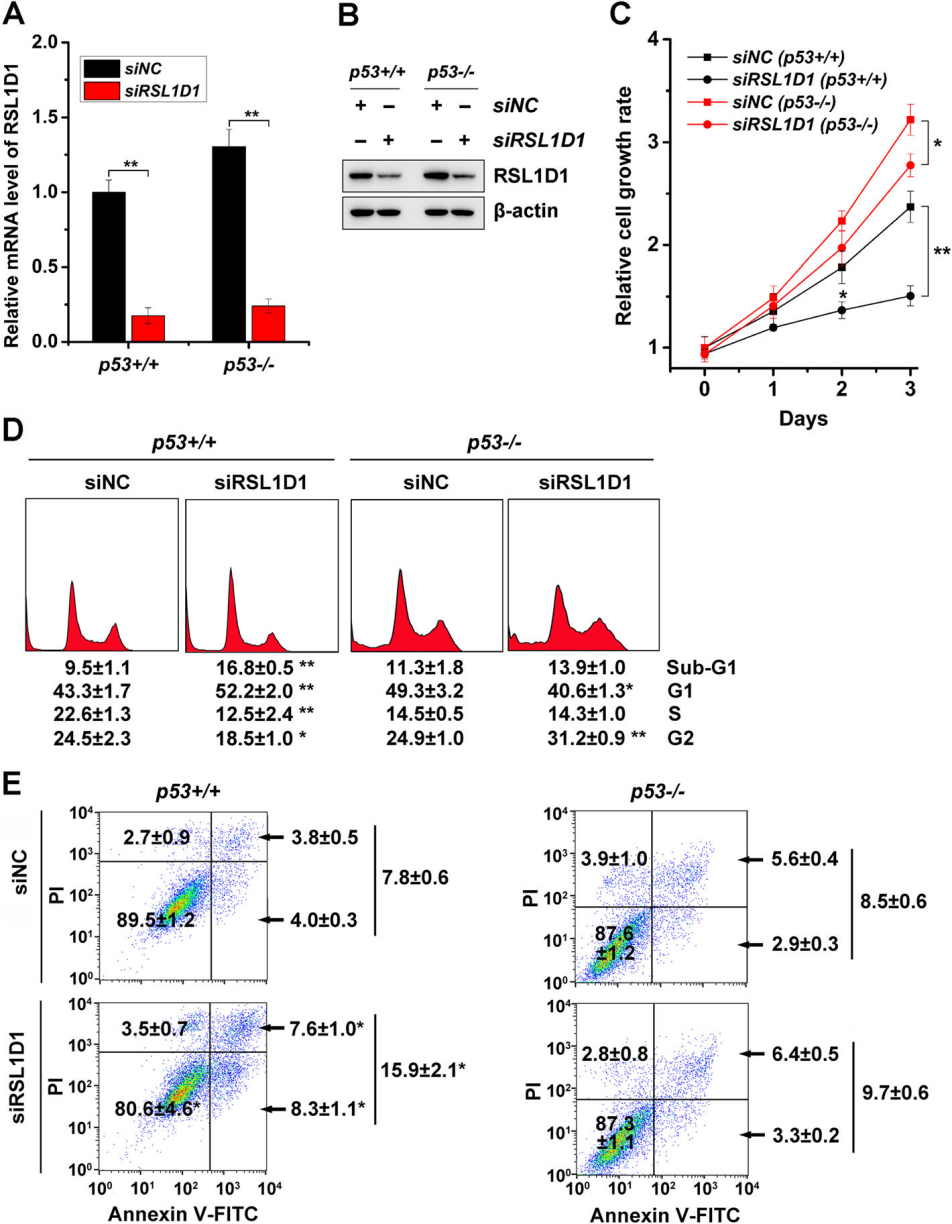
RSL1D1 Regulates the Proliferation and Survival of HCT116 Colorectal Cancer Cells. HCT116^*p53+/+*^ and HCT116^*p53−/−*^ cells were transfected with siRSL1D1 to downregulate RSL1D1 expression. The cells transfected with siNC were used as a negative control. **A** The mRNA levels of RSL1D1 were determined by qRT-PCR analysis. GAPDH was used as an internal control to normalize the values. The normalized value of siNC-treated HCT116^*p53+/+*^ cells was set to 1. **B** The levels of RSL1D1 protein were determined by western blot analysis. β-actin was used as a loading control. **C** Cell proliferation was evaluated by MTT assay. The cells were seeded to a 96-well plate and incubated for 0, 1, 2, and 3 days. The values on day 0 were normalized to 1. **D** Cell cycle was determined by PI staining. The stained cells were subjected to flow cytometry to analyze the average percentage of each cell cycle phase. Sub-G_1_ indicates the apoptotic cell population. **E** Cell apoptosis was determined by Annexin V-FITC/PI double staining. The stained cells were subjected to flow cytometry to analyze the percentage of apoptotic cells. The cells in the right lower (Annexin V-FITC^+^/PI^−^) and right upper (Annexin V-FITC^+^/PI^+^) quadrants indicate early and late apoptosis, respectively. **A**, **C**-**E** Data are represented as mean ± SD. Student’s t test. *P < 0.05 and **P < 0.01 denote significant difference

Hence, we transfected HCT116 cells with the siRSL1D1 to downregulate the expression of RSL1D1 and assess whether RSL1D1 is involved in cell proliferation. Efficient downregulation of RSL1D1 (approximately 80% at the mRNA level) (Fig. 1A and B) greatly slowed down cell proliferation either in the presence (*P* < 0.01) or absence (*P* < 0.05) of p53 (Fig. 1C). Three days after transfection with the siRSL1D1, HCT116^*p53+/+*^ and HCT116^*p53−/−*^ cells displayed a remarkable decrease in the proliferation rate by approximately 37 and 14%, respectively (Fig. 1C). Interestingly, even though RSL1D1 knockdown inhibited the proliferation of *p53−/−* cancer cells, the presence of p53 greatly enhanced the inhibitory effect (Fig. 1C). These findings indicate that RSL1D1 regulates cancer cell proliferation both in a p53-dependent and -independent manner. Since p53 is critical in cell cycle progression [34], RSL1D1 is probably involved in p53-mediated cell cycle control, thereby regulating the proliferation of *p53+/+* cells.

To test whether RSL1D1 affects cell cycle progression, we performed a PI staining assay to analyze the effect of RSL1D1 knockdown on the distribution of each cell cycle phase. In HCT116^*p53−/−*^ cells, downregulation of RSL1D1 resulted in a higher percentage of the G_2_ population and a lower percentage of the G_1_ population (*P* < 0.05), but had little effect on the percentage of either sub-G_1_ or S population (Fig. 1D), demonstrating that RSL1D1 knockdown induces G_2_ arrest in the absence of p53. In HCT116^*p53+/+*^ cells, downregulation of RSL1D1 led to a higher percentage of the sub-G_1_ (apoptotic) and G_1_ populations, but a lower percentage of the S and G_2_ populations (*P* < 0.05) (Fig. 1D), indicating that RSL1D1 knockdown induces cell apoptosis and G_1_ arrest, a typical feature of senescent cells, in a p53-dependent manner [19, 35, 36].

To further confirm the regulation of apoptosis by RSL1D1, we performed an Annexin V-FITC/PI double staining assay. The results showed that downregulation of RSL1D1 remarkably induced both early (Annexin V-FITC^+^/PI^−^) and late (Annexin V-FITC^+^/PI^+^) apoptosis in HCT116^*p53+/+*^ (*P* < 0.05) other than HCT116^*p53−/−*^ cells (Fig. 1E), indicating that RSL1D1 regulates apoptosis in a p53-dependent manner.

Collectively, RSL1D1 promotes cancer cell proliferation and survival and the status of p53 determines how RSL1D1 regulates these cellular processes. In the absence of p53, RSL1D1 facilitates the G_2_/M transition. In the presence of p53, the function of RSL1D1 shifts to inhibit apoptosis and facilitate the G_1_/S transition.

### RSL1D1 negatively regulates the protein level of nuclear p53

**Fig. 2:**
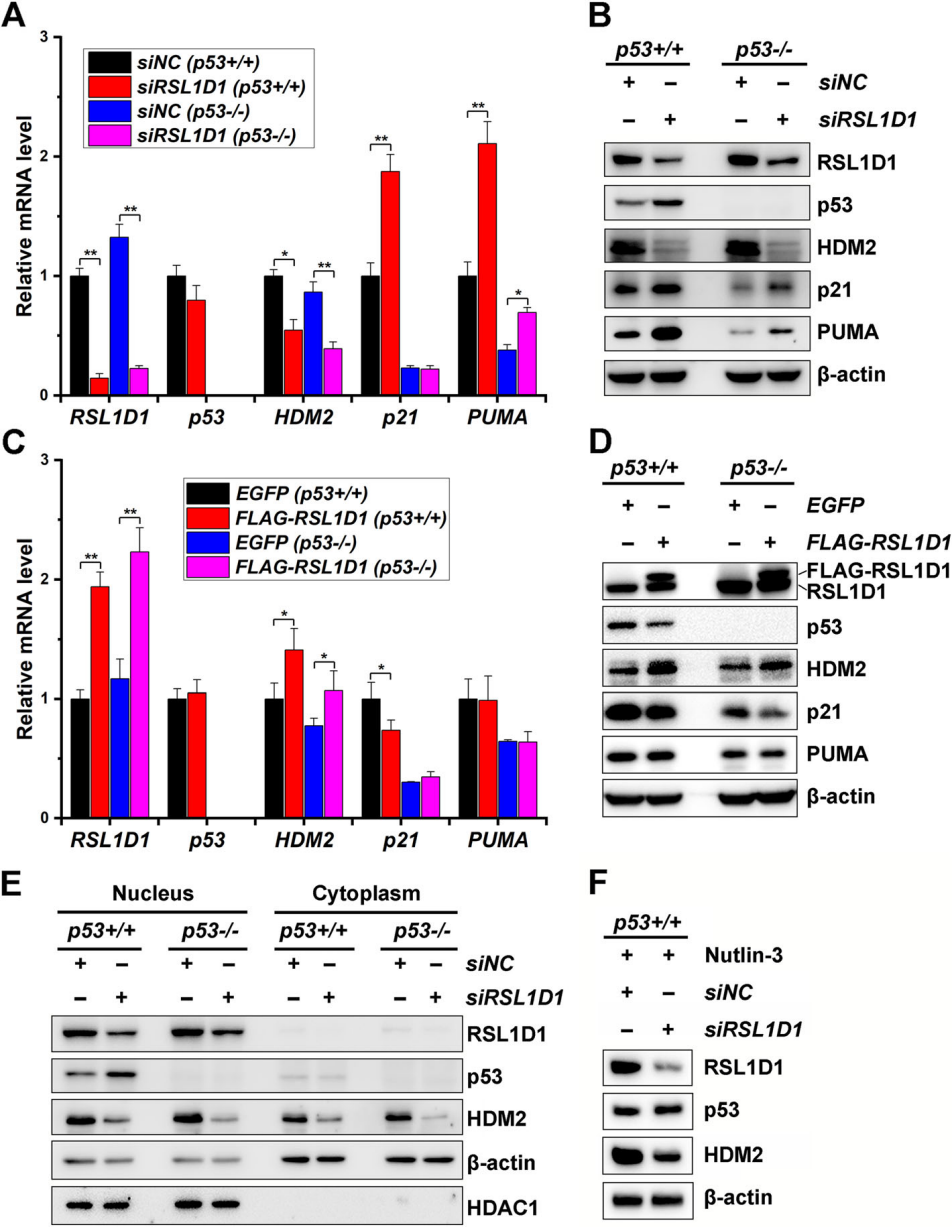
RSL1D1 Negatively Regulates the Level of Nuclear p53 Protein in an HDM2-Dependent Manner. Cells were transfected with siRSL1D1 to downregulate RSL1D1. For overexpression of RSL1D1, cells were transduced with lentiviruses inducibly expressing RSL1D1 and then treated with 1 μg/mL doxycycline for 72 h to induce expression. **A** The mRNA levels of RSL1D1, p53, HDM2, p21, and PUMA were determined by qRT-PCR analysis in RSL1D1-downregulated HCT116^*p53+/+*^ and HCT116^*p53−/−*^ cells. GAPDH was used as an internal control to normalize the values. The normalized values of siNC-transfected HCT116^*p53+/+*^ cells were set to 1. **B** The protein levels of RSL1D1, p53, HDM2, p21, and PUMA were determined by western blot analysis in RSL1D1-downregulated HCT116^*p53+/+*^ and HCT116^*p53−/−*^ cells. β-actin was set as a loading control. **C** The mRNA levels of RSL1D1, p53, HDM2, p21, and PUMA were determined by qRT-PCR in RSL1D1-overexpressed HCT116^*p53+/+*^ and HCT116^*p53−/−*^ cells. GAPDH was used as an internal control to normalize the values. The normalized values of EGFP-overexpressed HCT116^*p53+/+*^ cells were set to 1. **D** The protein levels of RSL1D1, p53, HDM2, p21, and PUMA were determined by western blot analysis in RSL1D1-overexpressed HCT116^*p53+/+*^ and HCT116^*p53−/−*^ cells. β-actin was used as a loading control. **E** The levels of nuclear and cytoplasmic RSL1D1, p53 and HDM2 proteins were determined by western blot analysis in RSL1D1-downregulated HCT116^*p53+/+*^ and HCT116^*p53−/−*^ cells. HDAC1 and β-actin were set as internal controls for nuclear and cytoplasmic proteins, respectively. **F** The levels of RSL1D1, p53, and HDM2 proteins were determined by western blot analysis in RSL1D1-downregulated HCT116^*p53+/+*^ cells treated with Nutlin-3 (40 μM, 12 h). β-actin was used as a loading control. **A**, **C** Data are represented as mean ± SD. Student’s t test. *P < 0.05 and **P < 0.01 denote significant difference

To investigate how RSL1D1 participates in the p53 signaling pathway, we modulated the expression of RSL1D1 in human CRC cells and analyzed the mRNA and protein levels of p53. The mRNA level of p53 showed no significant change in either RSL1D1-downregulated or -overexpressed HCT116^*p53+/+*^ cells when compared with that in the negative controls (Fig. 2A and C). However, the mRNA level of p21 increased remarkably in RSL1D1-downregulated cells (*P* < 0.01) and decreased in RSL1D1-overexpressed cells (*P* < 0.05) (Fig. 2A and C). Since p21 is a canonical target of p53 and can be induced by this transcription factor to arrest the cell cycle at the G1/S checkpoint [37, 38], RSL1D1 is likely to negatively regulate the level of p53 protein but not p53 mRNA. We therefore analyzed the protein levels of p53 and p21 in RSL1D1-modulated HCT116^*p53+/+*^ cells. The result showed that the protein levels of p53 and p21 increased in RSL1D1-downregulated HCT116^*p53+/+*^ cells (Fig. 2B), thereby inducing G1/S arrest (Fig. 1D). When RSL1D1 was overexpressed, the protein levels of p53 and p21 decreased (Fig. 2D).

Interestingly, RSL1D1 could negatively regulate p21 protein in a p53-independent manner (Fig. 2A-D). The protein level of p21 increased in response to RSL1D1 knockdown and decreased when RSL1D1 was overexpressed in HCT116^*p53−/−*^ cells (Fig. 2B and D). However, the mRNA level of p21 showed no significant change when RSL1D1 was either downregulated or upregulated in the absence of p53 (Fig. 2A and C). It is noteworthy that the protein level of p21 in siRSL1D1-transfected HCT116^*p53−/−*^ cells was still lower than that in siNC-transfected HCT116^*p53+/+*^ cells (Fig. 2B), accordingly insufficient to induce G1 arrest in the lack of the dominant contribution of p53 (Fig. 1D) [39, 40].

Furthermore, RSL1D1 could negatively regulate PUMA, another p53 target gene and a major apoptosis-inducing factor [41]. In the presence of p53, the mRNA (*P* < 0.01) and protein levels of PUMA increased significantly in RSL1D1-downregulated HCT116 cells (Fig. 2A and B), thereby inducing apoptosis in a p53-dependent manner (Fig. 1D and E). In the absence of p53, RSL1D1 knockdown also upregulated the mRNA (*P* < 0.05) and protein levels of PUMA (Fig. 2A and B). However, the level of PUMA expression in siRSL1D1-transfected HCT116^*p53−/−*^ cells was still lower than that in siNC-transfected HCT116^*p53+/+*^ cells (Fig. 2A and B), accordingly insufficient to induce apoptosis (Fig. 1D and E). To investigate how RSL1D1 regulates PUMA expression in HCT116^*p53−/−*^ cells, we determined the protein level of FOXO3a, a direct transcriptional regulator of PUMA that mainly contributes to the p53-independent upregulation of PUMA in CRC cells [42, 43]. Upon RSL1D1 knockdown, the level of FOXO3a protein increased in HCT116^*p53−/−*^ other than HCT116^*p53+/+*^ cells (Supplementary Fig. S2A). Further study showed that FOXO3a knockdown decreased the high level of PUMA expression in RSL1D1-downregulated HCT116^*p53−/−*^ cells (Supplementary Fig. S2B). These data demonstrate that RSL1D1 knockdown increases PUMA expression by upregulation of FOXO3a in the absence of p53. The upregulation of FOXO3a contributed to G2/M arrest in RSL1D1-downregulated HCT116^*p53−/−*^ cells, thereby inhibiting cell proliferation (Fig. 1C and D), which is consistent with the current opinion that FOXO3a activation induces G2/M arrest in various cancer cells [44–47]. However, the mRNA and protein levels of PUMA showed no significant change when RSL1D1 was overexpressed in either *p53+/+* or *p53−/−* CRC cells (Fig. 2C and D).

To further confirm the negative regulation of p53 by RSL1D1, we also evaluated the mRNA and protein levels of p53 and its target genes in RSL1D1-downregulated HCT-8 cells, another human CRC cell line harboring wild-type p53 [48]. Similarly, the mRNA level of p53 showed no significant change. In contrast, the mRNA levels of p21 and PUMA increased remarkably in HCT-8 cells in response to RSL1D1 knockdown (*P* < 0.05) (Supplementary Fig. S3A). However, the protein levels of p53, p21, and PUMA were all significantly increased (Supplementary Fig. S3B). Again, the data from HCT-8 cells support our hypothesis.

As a transcription factor, p53 is mainly localized in the nucleus and binds to the upstream activating sequences of target genes, such as p21 and PUMA, for transcriptional activation, leading to growth inhibition and apoptosis of cancer cells [49, 50]. To investigate whether RSL1D1 negatively regulates p53 in the nucleus, we separated the nucleus and cytoplasm from RSL1D1-downregulated HCT116 cells and measured the levels of p53 protein in these two subcellular compartments. Western blot analysis showed that in normal HCT116^*p53+/+*^ cancer cells, the level of p53 protein in the cytoplasm was much lower than that in the nucleus (Fig. 2E). When RSL1D1 expression was silenced by transfection with the siRSL1D1, the level of p53 protein increased significantly in the nucleus, but not in the cytoplasm (Fig. 2E).

Taken together, RSL1D1 negatively regulates the protein level of nuclear p53, thereby suppressing p53 targets to promote the proliferation and survival of CRC cells.

### RSL1D1 promotes p53 ubiquitination by upregulating HDM2

**Fig. 3:**
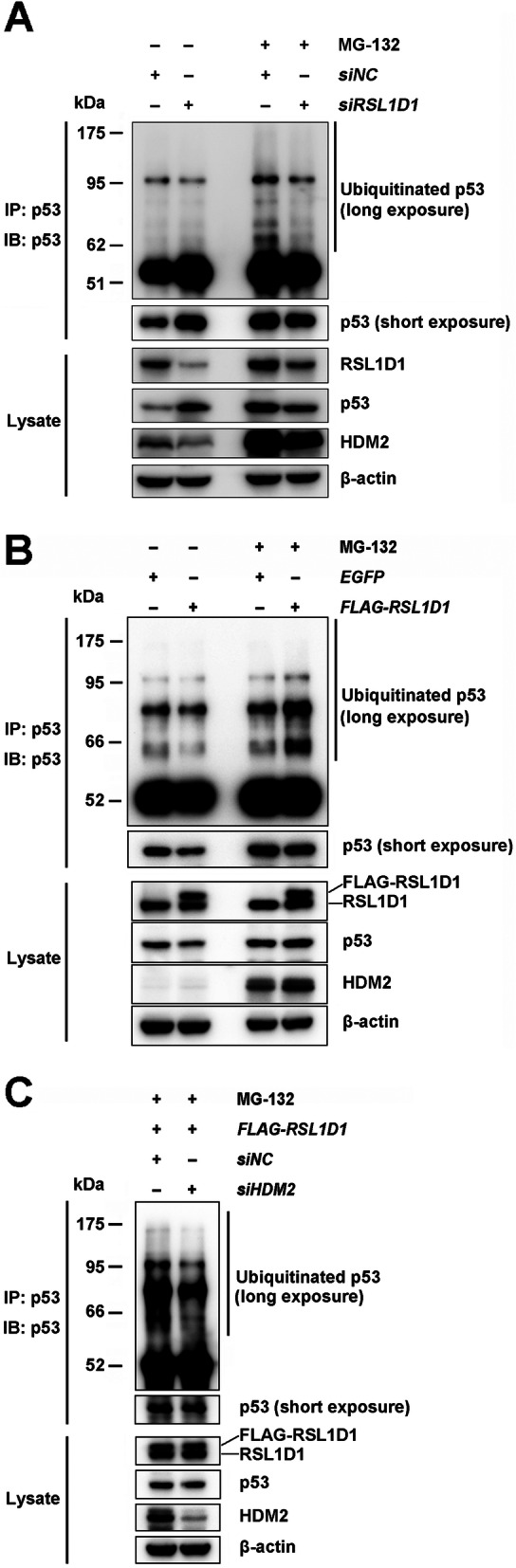
RSL1D1 Promotes p53 Ubiquitination by Upregulating HDM2. RSL1D1-downregulated or -overexpressed HCT116^*p53+/+*^ cells was treated with MG-132 (25 μM) for 6 h to inhibit the degradation of ubiquitinated p53. Monoclonal antibody against p53 (DO-1) was used for IP and western blot analyses of ubiquitinated and non-ubiquitinated p53. **A** The levels of ubiquitinated p53 proteins were evaluated in siRSL1D1- or siNC-transfected HCT116^*p53+/+*^ cells. The levels of RSL1D1, p53, and HDM2 proteins in the input cell lysate were determined by western blot analysis and β-actin was used as a loading control. **B** The levels of ubiquitinated p53 proteins were evaluated in RSL1D1- or EGFP-overexpressed HCT116^*p53+/+*^ cells. The levels of RSL1D1, p53, and HDM2 proteins in the input cell lysate were determined by western blot analysis and β-actin was used as a loading control. **C** The levels of ubiquitinated p53 proteins were evaluated in RSL1D1-overexpressed HCT116^*p53+/+*^ cells transfected with siHDM2 or siNC. The protein levels of RSL1D1, p53, and HDM2 in the input cell lysate were determined by western blot analysis and β-actin was used as a loading control

Since ubiquitination plays a major part in the negative regulation of p53 by acting as a signal for proteasome-mediated degradation [51], we wondered whether RSL1D1 is involved in ubiquitin-mediated p53 degradation and therefore analyzed p53 ubiquitination in *RSL1D1*-modulated HCT116^*p53+/+*^ cells treated with the proteasome inhibitor MG-132 [20]. The result showed that downregulation of RSL1D1 significantly decreased the amount of ubiquitinated p53 (Fig. 3A). In contrast, overexpression of RSL1D1 increased ubiquitinated p53 remarkably (Fig. 3B).

As an E3 ubiquitin ligase, HDM2 binds, ubiquitinates, and thereby negatively regulates p53 [52–54]. To test whether RSL1D1 promotes p53 ubiquitination by upregulating HDM2, we assessed the expression of HDM2 in RSL1D1-modulated cells. Compared with the controls, downregulation of RSL1D1 significantly decreased the mRNA (*P* < 0.05) and protein levels of HDM2 (Fig. 2A and B) and overexpression of this gene remarkably increased the expression of HDM2 (Fig. 2C and D) in either HCT116^*p53+/+*^ or HCT116^*p53−/−*^ cells, indicating that RSL1D1 positively regulates HDM2 expression in a p53-independent manner. Furthermore, the p53-independent upregulation of HDM2 by RSL1D1 also increased the protein levels of p21 and FOXO3a in RSL1D1-downregulated HCT116^*p53−/−*^ cells (Fig. 2B and Supplementary Fig. S2A), which is consistent with the current opinion that HDM2 directly interacts with p21 and FOXO3a for ubiquitination [55–57], thereby negatively regulating the levels of these two proteins. In *p53+/+* cells, the RSL1D1-HDM2 signaling axis majorly contributed to downregulation of p53 (Fig. 2), but had no effect on the FOXO3a level (Supplementary Fig. S2A). However, in *p53−/−* cells, its function shifts to negatively regulate FOXO3a (Supplementary Fig. S2A).

To test whether the negative regulation of p53 by RSL1D1 is HDM2-dependent, we treated HCT116^*p53+/+*^ cells with Nutlin-3 to block p53-HDM2 interaction [58]. Unlike in the untreated cells (Fig. 2B), RSL1D1 knockdown did not affect the protein level of p53 in Nutlin-3 treated cells, but still decreased the protein level of HDM2 (Fig. 2F). This indicates that RSL1D1 negatively regulates p53 in a HDM2-dependent manner.

Since HDM4 (or HDMX) is also a negative regulator of p53 in the regulatory feedback loop of nucleolar protein-HDM2-p53 [59, 60], we wondered whether HDM4 is also involved in the regulation of p53 by RSL1D1. The result showed that the protein levels of HDM4 were not remarkably affected by RSL1D1 knockdown in either HCT116^*p53+/+*^ or HCT116^*p53−/−*^ cells (Supplementary Fig. S4). It is unlikely that RSL1D1 affects p53 levels via HDM4.

To further confirm that RSL1D1 promotes HDM2-mediated p53 ubiquitination, we assessed the level of ubiquitinated p53 protein in HDM2-downregulated, RSL1D1-overexpressed HCT116^*p53+/+*^ cells. The result showed that HDM2 knockdown significantly decreased p53 ubiquitination in RSL1D1-overexpressed cells (Fig. 3C), indicating that HDM2 locates downstream of RSL1D1 in the signaling axis of p53 ubiquitination.

To rule out the possibility that HDM2 regulates RSL1D1, we assessed the expression of RSL1D1 in HDM2-downregulated HCT116^*p53+/+*^ cells. As expected, HDM2 knockdown did not change the level of p53 mRNA, but remarkably increased the amount of p53 protein, thereby upregulating the protein and mRNA levels of p21 and PUMA (*P* < 0.01) (Supplementary Fig. S5). However, downregulation of HDM2 did not significantly change the mRNA or protein levels of RSL1D1 (Supplementary Fig. S5), indicating that RSL1D1 locates upstream of HDM2 in the RSL1D1-HDM2 signaling axis.

Collectively, RSL1D1 is an upstream factor in the RSL1D1-HDM2 signaling axis and positively regulates HDM2 to promote p53 ubiquitination.

### RSL1D1 upregulates HDM2 by stabilizing HDM2 mRNA

**Fig. 4:**
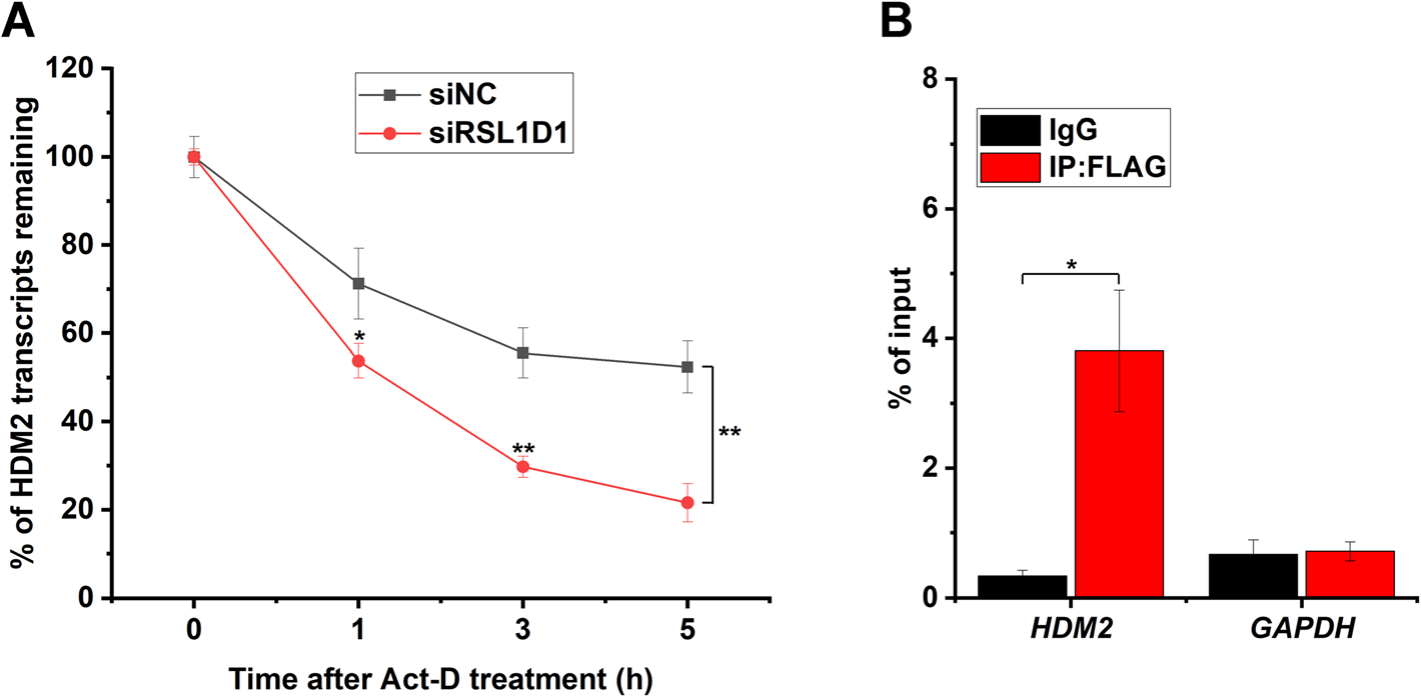
RSL1D1 Interacts with HDM2 mRNA to Increase Its Stability. **A** Downregulation of RSL1D1 increases the decay rate of HDM2 transcripts. siRNA-transfected HCT116^*p53−/−*^ cells were treated with 4 μM Act-D for 0, 1, 3, and 5 h. The mRNA level of HDM2 was determined by qRT-PCR analysis. GAPDH was used as an internal control to normalize the values. The normalized values of siNC- or siRSL1D1-transfected cells at 0 h were set to 100%. **B** RIP assay was performed to evaluate the interaction between RSL1D1 and HDM2 mRNA. Anti-FLAG antibody was used for RIP utilizing HCT116^*p53−/−*^ cells overexpressing FLAG-RSL1D1. Mouse IgG was used as a negative control. The mRNA levels of HDM2 and GAPDH were determined by qRT-PCR. The mRNA levels of input were used to normalize the values. **A**, **B** Data are represented as mean ± SD. Student’s t test. *P < 0.05 and **P < 0.01 denote significant difference

As a canonical p53 target, HDM2 is upregulated upon p53 activation, which in return inhibits p53 [61]. However, in the current study, the mRNA level of HDM2 decreased in response to a high level of p53 protein in RSL1D1-downregulated HCT116 cells (Fig. 2A). Since RSL1D1 participates in the regulation of mRNA stability of PTEN and NOLC1 via protein-RNA interaction [19, 21], RSL1D1 is also possibly involved in regulating the stability of HDM2 mRNA. To verify this, we first evaluated the stability of HDM2 mRNA in siRSL1D1-transfected HCT116 cells. Compared with the controls, downregulation of RSL1D1 remarkably accelerated the degradation of HDM2 transcripts (Fig. 4A), indicating that RSL1D1 is an important factor in maintaining the stability of HDM2 mRNA.

To explore whether RSL1D1 stablizes HDM2 mRNA via protein-RNA interaction, we performed an RIP assay and determined the amount of HDM2 mRNA in the immunoprecipitate from FLAG-RSL1D1-overexpressed cells. Compared with the negative controls, HDM2 transcripts were significantly precipitated by anti-FLAG antibody (*P* < 0.05) (Fig. 4B), demonstrating that RSL1D1 interacts with HDM2 mRNA.

### RSL1D1 Colocalizes with p53 and HDM2 in the nucleus of colorectal Cancer cells

**Fig. 5:**
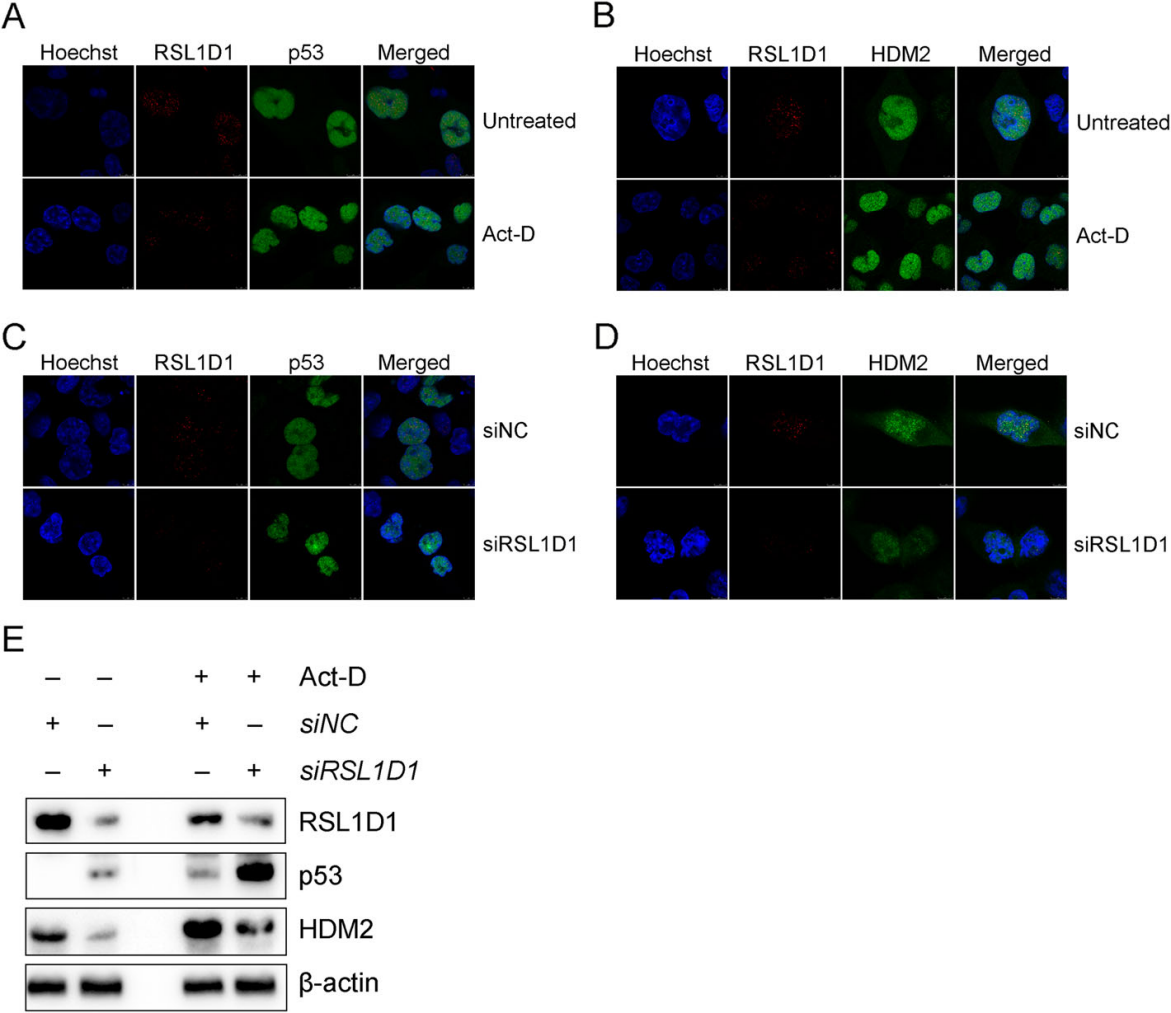
RSL1D1 Colocalizes with p53 and HDM2 and Negatively Regulates p53 in HCT116^*p53+/+*^ Cells under Normal and Nucleolar Stress Conditions. **A**, **B** IFA was performed to investigate the sub-cellular localization of RSL1D1 (red) (**A**, **B**), p53 (green) (**A**), and HDM2 (green) (**B**) in HCT116^*p53+/+*^ cells treated or untreated with 5 nM Act-D for 24 h. **C**, **D** IFA was performed to investigate the sub-cellular localization of RSL1D1 (red) (**C**, **D**), p53 (green) (**C**), and HDM2 (green) (**D**) in the siRSL1D1- or siNC-transfected HCT116^*p53+/+*^ cells. **A**-**D** The nuclei were stained with Hoechst (blue). Yellow color indicates the colocalization of RSL1D1 either with p53 (**A**, **C**) or HDM2 (**B**, **D**). Scale bars: 5 μm. **E** The levels of RSL1D1, p53, and HDM2 proteins were measured by western blot analysis in HCT116^*p53+/+*^ cells transfected with siRSL1D1 or siNC. The cells were treated or untreated with 5 nM Act-D for 24 h. β-actin was set as a loading control

Since RSL1D1, as a nucleolus-localized protein, is released to the nucleoplasm of H1299 non-small cell lung cancer cells upon nucleolar stress [20], an IF assay was performed to address whether this nucleolus-nucleoplasm translocation of RSL1D1 occurs in HCT116 CRC cells. We first assessed the applicability of homemade anti-RSL1D1 monoclonal antibody to IFA. Compared with the negative controls, the antibody led to a weaker staining in RSL1D1-downregulated HCT116^*p53+/+*^ cells and a stronger staining in RSL1D1-overexpressed cells (Supplementary Fig. S6), indicating that the homemade antibody is specific to RSL1D1. Unlike in H1299 cells, RSL1D1 was not limited to the nucleolus but distributed throughout the entire nucleus and colocalized with p53 or HDM2 in HCT116^*p53+/+*^ cells under normal conditions (Fig. 5A and B). Upon Act-D-induced nucleolar stress [20], the intranuclear distribution and colocalization of RSL1D1, HDM2, and p53 remained unchanged (Fig. 5A and B), even though the levels of these three proteins changed (Fig. 5E). Moreover, downregulation of RSL1D1 also did not change the overall distribution of RSL1D1, HDM2, and p53 (Fig. 5C and D). Furthermore, to explore whether nucleolar stress affects the role of RSL1D1 in the regulation of p53 and HDM2, we determined the protein levels of p53 and HDM2 in RSL1D1-downregulated HCT116^*p53+/+*^ cells untreated or treated with Act-D. The results showed that RSL1D1 knockdown reduced HDM2 expression and thereby upregulated p53 regardless of the Act-D treatment (Fig. 5E).

Collectively, RSL1D1 colocalizes with p53 and HDM2 in the nucleus of CRC cells and nucleolar stress does not affect the overall distribution and function of RSL1D1 as a regulator of p53 and HDM2, which probably contributes to the negative regulation of p53 by RSL1D1 under various conditions.

### RSL1D1 recruits p53 to HDM2 for ubiquitination

**Fig. 6:**
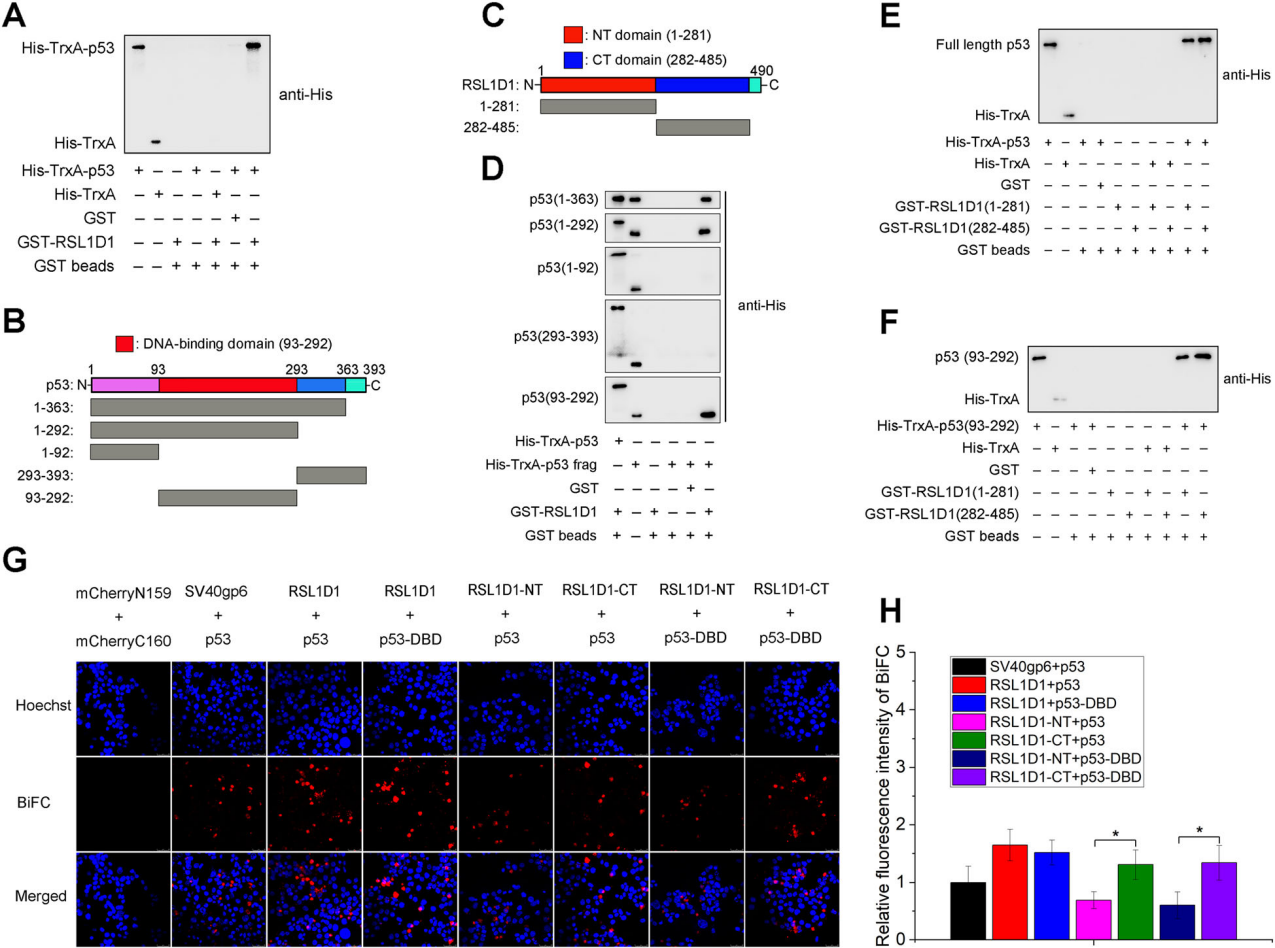
RSL1D1 Directly Interacts with p53. **A** GST pull-down assays were performed to evaluate the interaction between full-length RSL1D1 (RSL1D1-FL) and p53 (p53-FL) in vitro. RSL1D1-FL and p53-FL were GST and His tagged, respectively. **B**, **C** Schematic diagrams showing p53 (**B**), RSL1D1 (**C**), and their truncated variants constructed in this study. **D**, **E** GST pull-down assays were performed to map the RSL1D1-binding domain on p53 (**D**) and the p53-binding domain on RSL1D1 (**E**). RSL1D1-FL and its truncated variants (RSL1D1-NT and RSL1D1-CT) were GST tagged, whereas p53-FL and its truncated variants were His tagged. **F** GST pull-down assays were carried out to evaluate the interaction between the RSL1D1-binding domain on p53 and the p53-binding domains on RSL1D1. **G** Bimolecular fluorescence complementation (BiFC) assay was performed to confirm the interaction between RSL1D1 and p53 in vivo. RSL1D1-FL, RSL1D1-NT, and RSL1D1-CT were cloned into pBiFC-mCherryN159, whereas p53-FL and p53-DBD were cloned into pBIFC-mCherryC160. The recombinant plasmid pairs were co-transfected into HCT116^*p53+/+*^ cells. The in vivo interaction between two proteins fused with mCherryN159 and mCherryC160, respectively, was indicated by the red fluorescence in the cells and the nucleus was stained by Hoechst (blue). Co-transfection with empty plasmids pBiFC-mCherryN159 and pBiFC-mCherryC160 was set as a negative control, whereas co-transfection with plasmids pBiFC-mCherryN159-SV40gp6 and pBiFC-mCherryC160-p53 was used as a positive control. Scale bars: 50 μm. **H** The relative fluorescence intensity in different BiFC groups. To facilitate comparison, the mean value of fluorescence intensity in the cells co-transfected with pBiFC-mCherryN159-SV40gp6 and pBiFC-mCherryC160-p53 was set to 1. Data are represented as mean ± SD. Student’s t test. *P < 0.05 and **P < 0.01 denote significant difference

**Fig. 7:**
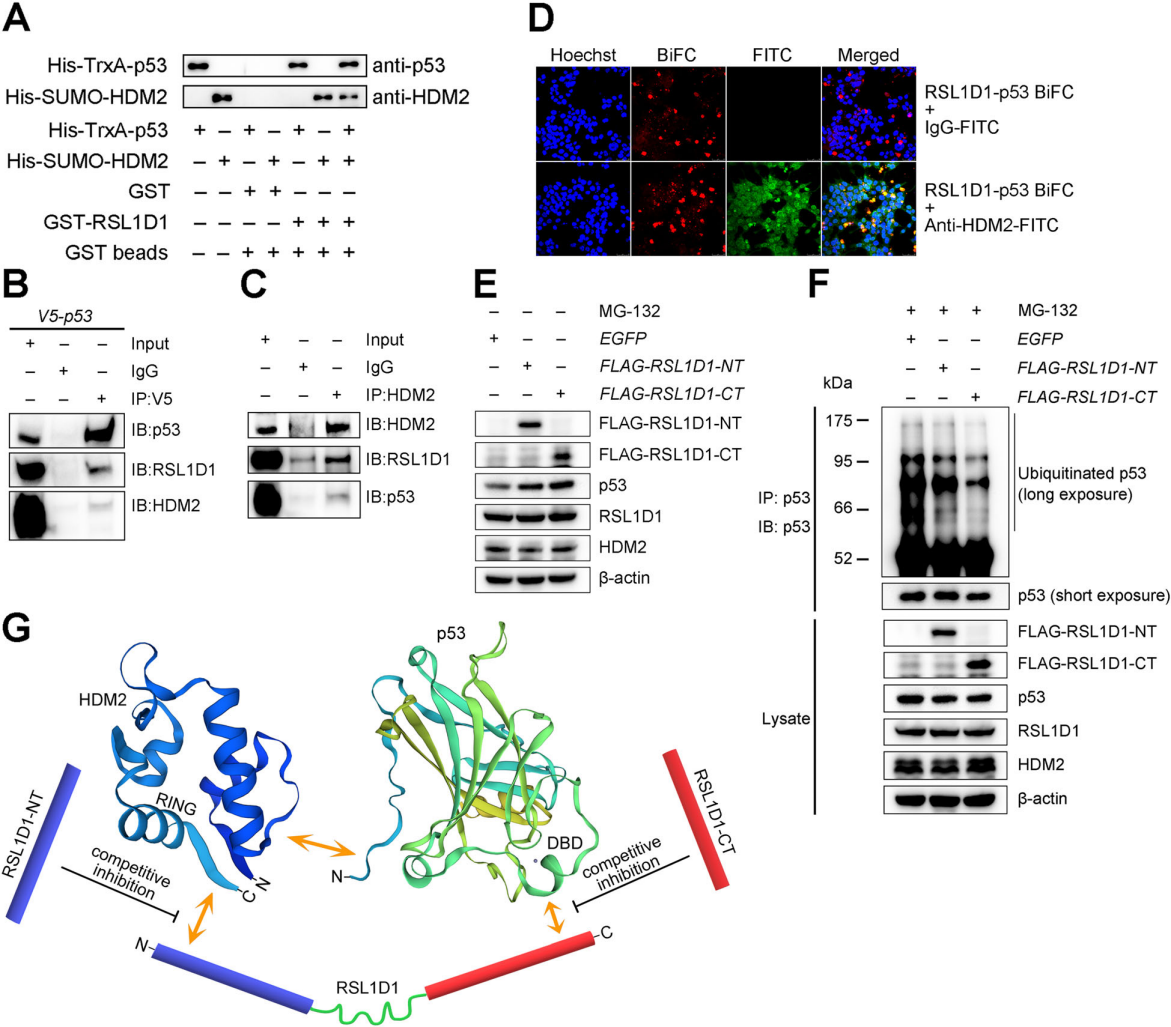
RSL1D1 Recruits p53 to HDM2 to Enhance p53 Ubiquitination. **A** GST pull-down assays were performed to evaluate the interaction between RSL1D1, p53, and HDM2 in vitro. RSL1D1 was GST-tagged, whereas p53 and HDM2 were His-tagged. **B**, **C** Co-IP assay was performed to evaluate the interaction between RSL1D1, p53, and HDM2 in vivo. Lentivirus-transduced HCT116^*p53−/−*^ cells stably expressing V5-p53 (**B**) or HCT116^*p53+/+*^ cells (**C**) were harvested and the lysate was immunoprecipitated with anti-V5 (**B**) or anti-HDM2 (**C**) antibody. Rabbit IgG was used as a negative control. Input represents 2.5% (**B**) or 5% (**C**) of the lysate utilized for IP. **D** A combination of BiFC and IF assays was performed to evaluate the intracellular co-localization of RSL1D1, p53, and HDM2. After co-transfection with pBiFC-mCherryN159-RSL1D1 and pBiFC-mCherryC160-p53, HCT116 cells were incubated with anti-HDM2 antibody and then FITC-labeled anti-IgG. Red fluorescence indicates an RSL1D1-p53 complex. Green fluorescence indicates HDM2 protein. Yellow color indicates a ternary protein complex comprising RSL1D1, p53, and HDM2. The nuclei were stained with Hoechst (blue). Scale bars: 50 μm. **E** The protein levels of p53, RSL1D1, and HDM2 were determined in HCT116^*p53+/+*^ cells overexpressing truncated RSL1D1 variants or EGFP. The cells were transduced with lentiviruses inducibly expressing genes of interest and treated with 1 μg/mL doxycycline to induce gene expression for 24 h. The protein levels of RSL1D1-NT, RSL1D1-CT, p53, RSL1D1 and HDM2 were determined by western blot analysis. β-actin was used as a loading control. **F** The levels of ubiquitinated p53 proteins were evaluated in HCT116^*p53+/+*^ cells overexpressing truncated RSL1D1 variants or EGFP. The cells were transduced with lentiviruses inducibly expressing genes of interest and treated with 1 μg/mL doxycycline to induce gene expression for 24 h, followed by an additional treatment with 25 μM MG-132 for 6 h. DO-1 was used for IP and western blot analyses of the ubiquitinated or non-ubiquitinated p53 protein. The protein levels of RSL1D1-NT, RSL1D1-CT, p53, RSL1D1, and HDM2 in the input lysate were determined by western blot analysis and β-actin was used as a loading control. **G** A model showing the interaction between RSL1D1, p53, and HDM2, which can be competitively destroyed by overexpression of either RSL1D1-NT or RSL1D1-CT

The N-terminus of HDM2 (aa 1–125) reportedly interacts with the transactivation domain of p53 (aa 15–29) [62]. Recently, Xie and colleagues have reported that the C-terminus of HDM2 (aa 349–489) interacts with RSL1D1 [20]. Considering the roles of RSL1D1 (Fig. 3) and HDM2 [52] in p53 ubiquitination and the colocalization of RSL1D1 with p53 and HDM2 (mainly in the nucleus) (Fig. 5), RSL1D1 might recruit p53 to HDM2 via direct interaction to form a transient ternary protein complex (Fig. 7G), thereby enhancing HDM2-mediated p53 ubiquitination. To address this, we first explored the possible interaction between RSL1D1 and p53. GST-pulldown and co-IP analyses showed that full-length p53 (p53-FL) interacted with full-length RSL1D1 (RSL1D1-FL) both in vitro and in vivo (Fig. 6A and 7B).

Then, a panel of truncated RSL1D1 and p53 variants was constructed to identify the RSL1D1-binding domain on p53 and the p53-binding domain on RSL1D1 (Fig. 6B and C). The recombinant proteins were purified using affinity chromatography (Supplementary Fig. S7A and B). GST-pulldown analysis showed that the DNA-binding domain of p53 (p53-DBD, aa 93–292) [63] interacted with the RSL1D1-FL (Fig. 6D). The N-terminus (RSL1D1-NT, aa 1–281) and C-terminus (RSL1D1-CT, aa 282–485) of RSL1D1 both interacted with the p53-FL (Fig. 6E), more accurately, the p53-DBD (Fig. 6F). Interestingly, compared with the RSL1D1-NT, the RSL1D1-CT interacted more strongly with the p53-FL and the p53-DBD in vitro (Fig. 6E and F).

To further confirm the RSL1D1-p53 interaction, we constructed a panel of BiFC plasmids expressing the p53-FL, the RSL1D1-FL, or their truncated variants. Plasmid pairs were co-transfected into HCT116 cells. Fluorescent images showed that both p53-FL and p53-DBD interacted with the RSL1D1-FL, the RSL1D1-NT, and the RSL1D1-CT in vivo (Fig. 6G). In agreement with the GST-pulldown data (Fig. 6E and F), the relative fluorescence intensity of BiFC (Fig. 6G and H) also indicated the preference of the p53-FL and the p53-DBD to bind the RSL1D1-CT other than the RSL1D1-NT which has been identified as the binding site for HDM2 [20].

To explore whether RSL1D1, HDM2, and p53 form a ternary protein complex, we evaluated the colocalization of these three proteins. GST-pulldown analysis showed that RSL1D1 interacted simultaneously with HDM2 and p53 in vitro (Fig. 7A and Supplementary Fig. S7). The result of co-IP also verified the RSL1D1-p53, RSL1D1-HDM2, and p53-HDM2 interactions in HCT116 cells (Fig. 7B and C). Then, we transfected HCT116^*p53+/+*^ cells with two BiFC plasmids expressing the RSL1D1-FL and p53-FL proteins, respectively, followed by an immunofluorescence assay against HDM2. The result revealed an obvious colocalization of RSL1D1, HDM2, and p53 (Fig. 7D). Together, these in vitro and in vivo data strongly suggest that RSL1D1, HDM2, and p53 form a transient ternary protein complex in HCT116 cells.

Next, to explore whether the p53-RSL1D1 interaction recruits p53 to HDM2 for ubiquitination, we performed a competitive ubiquitination assay using the RSL1D1-NT or the RSL1D1-CT as a competitive inhibitor. Compared with the EGFP control, overexpression of either RSL1D1-NT or RSL1D1-CT significantly increased the levels of p53 protein (Fig. 7E) by inhibition of p53 ubiquitination (Fig. 7F) in HCT116^*p53+/+*^ cells, consistent with the increased level of p53 protein (Fig. 2B) and decreased p53 ubiquitination (Fig. 3A) in RSL1D1-downregulated cells. These data indicate that RSL1D1 recruits p53 to HDM2 for ubiquitination and HDM2-mediated p53 ubiquitination can be alleviated by competitive occupation of the RSL1D1-binding site on p53 or HDM2 (Fig. 7G).

Collectively, RSL1D1 recruits p53 to HDM2 via protein-protein interactions to form a transient ternary protein complex, which enhances HDM2-mediated p53 ubiquitination.

### RSL1D1 is a potential molecular target for anti-tumor therapy

**Fig. 8:**
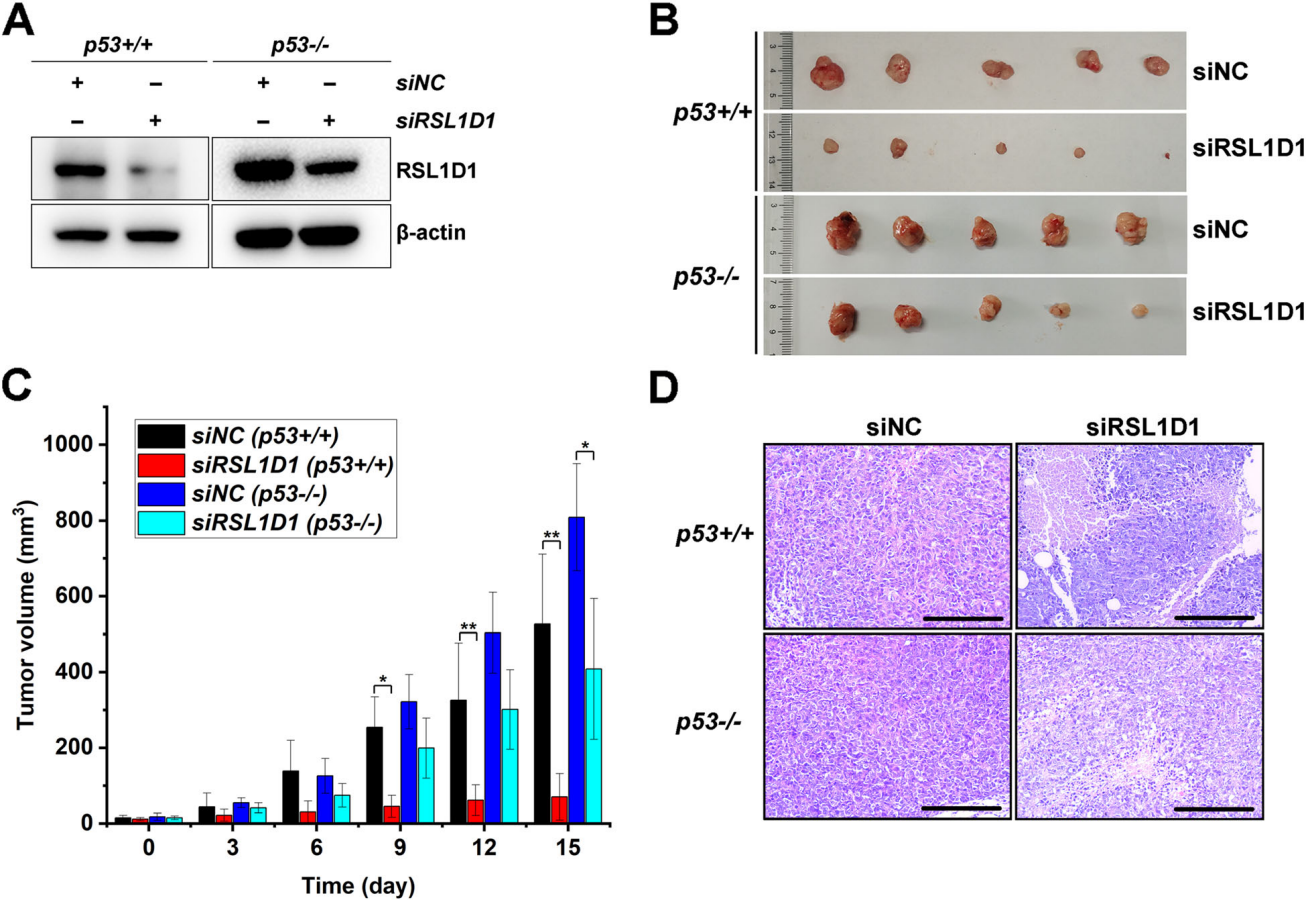
RSL1D1 Is a Potential Molecular Target for Colorectal Cancer Treatment. Nude mice bearing HCT116^*p53+/+*^ or HCT116^*p53−/−*^ tumors were randomly grouped and treated *diebus tertius* for 15 days with siNC or siRSL1D1 by intratumor transfection. **A** Western blot analysis of the amount of RSL1D1 protein in siRSL1D1- or siNC-treated tumors at the end of a 15-day treatment course. **B** Images of tumors from mice sacrificed at day 15. **C** Mean tumor volumes in each group during a 15-day treatment course. Data are represented as mean ± SD, *n* = 5. Student’s t test. *P < 0.05 and **P < 0.01 denote significant difference. **D** Representative images of tumor tissues revealed by H&E staining. Scale bars: 50 μm

To address whether RSL1D1 is a potential target in antitumor therapeutics, we evaluated the efficacy of the siRSL1D1 in treating HCT116^p53+/+^ or HCT116^*p53−/−*^ tumors in nude mice in a 15-day antitumor treatment. The intratumor levels of RSL1D1 protein were effectively downregulated by siRSL1D1 treatment in both *p53+/+* and *p53−/−* xenografts (Fig. 8A), leading to a significant inhibition of tumor growth (Fig. 8B and C). The siRSL1D1 group showed a reduction of approximately 90% in the mean volume of *p53+/+* tumors (*P* < 0.01), compared with approximately 50% in that of *p53−/−* tumors (*P* < 0.05). Similar to the in vitro data (Fig. 1C), the presence of p53 greatly enhanced the in vivo tumor inhibitory effect of siRSL1D1 treatment. These data demonstrate that RSL1D1 knockdown inhibits tumor growth both in a p53-dependent and -independent manner and p53 contributed to a major part of the efficacy in treating HCT116^*p53+/+*^ tumors (Fig. 8B and C).

To further evaluate the antitumor efficacy of the siRSL1D1, a histopathological analysis was performed. Compared with the siNC controls, siRSL1D1-treated *p53+/+* tumor tissues displayed cavitation and most tumor cells showed a hyperchromatic nucleus and condensed cytoplasm (Fig. 8D). These typical morphological features of cell apoptosis and necrosis, along with the decreased mean tumor volume (Fig. 8B and C), suggested a potent tumor-suppressive effect induced by siRSL1D1 treatment. Moreover, the histological data also revealed a tumor-suppressive effect of the siRSL1D1 on *p53−/−* tumors, but with a lower efficacy (Fig. 8D).

Taken together, RSL1D1 is a potential therapeutic target for CRC and downregulation of RSL1D1 is a highly efficient therapeutic strategy against HCT116^*p53+/+*^ tumors.

## Discussion

**Fig. 9:**
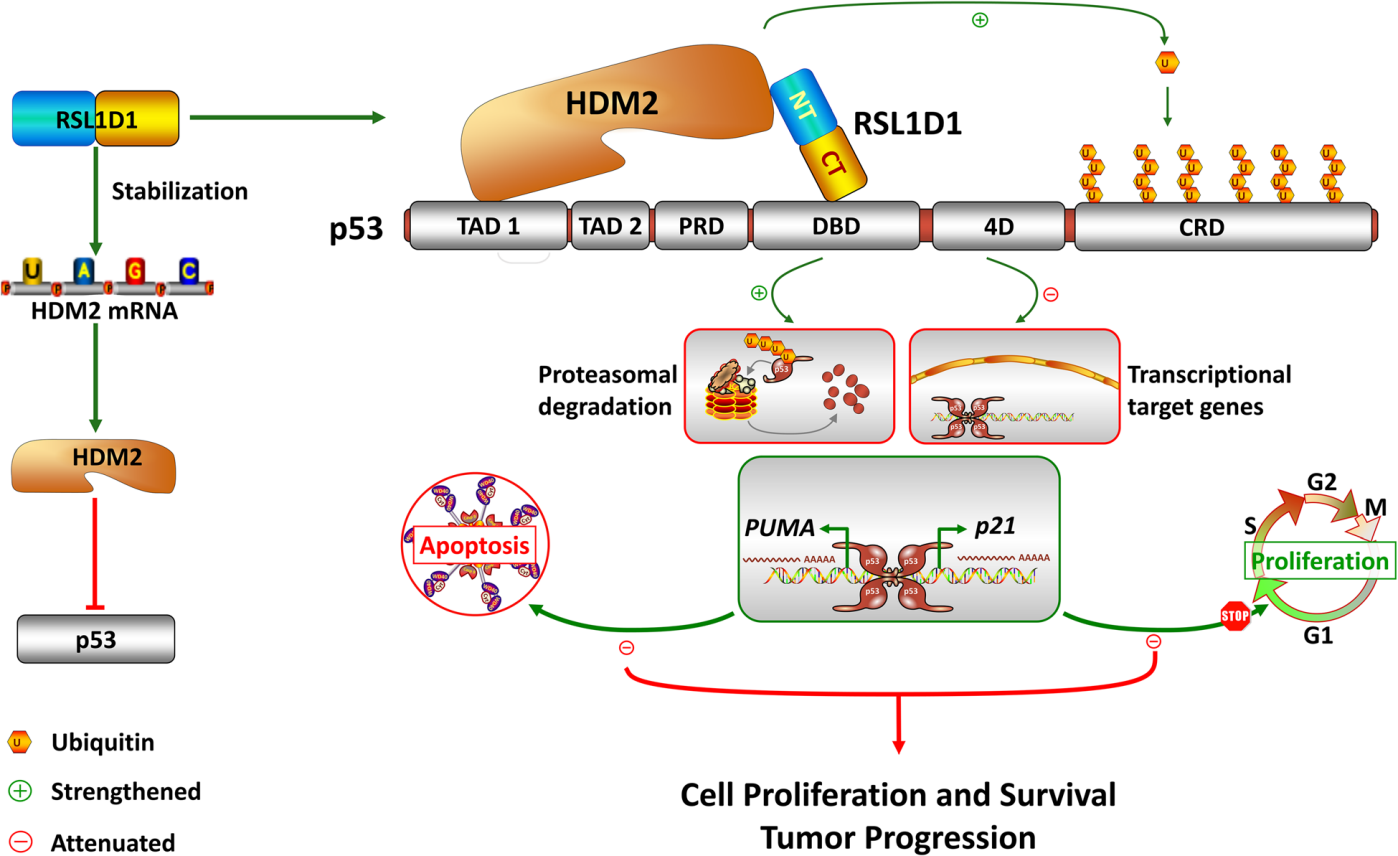
A Proposed Regulatory Triangle Model for Tumor Progression Involving RSL1D1, p53, and HDM2. In this model, RSL1D1 directly downregulates p53 by recruiting it to HDM2 to form a transient ternary protein complex, thereby enhancing HDM2-mediated p53 ubiquitination and degradation. RSL1D1 also indirectly downregulates p53 by stabilizing HDM2 mRNA to increase the protein level of HDM2. The ensuing low expression of p53 target genes, such as p21 and PUMA, facilitates the proliferation and survival of CRC cells, which is conducive to tumor progression

RSL1D1 is an important nucleolar protein to participate in multiple biological processes [22], such as cellular senescence [19, 64], cell migration and proliferation [16, 17], and cell apoptosis [15]. It has recently been reported that RSL1D1 translocates to the nucleoplasm in response to nucleolar stress, which contributes to the stabilization of p53 by inhibiting HDM2-mediated ubiquitination through direct interaction with the RING finger domain of HDM2 in U-2 OS and H1299 cells [20]. In contrast to this model, we discovered that RSL1D1 negatively regulates p53 by upregulating HDM2 and forming a ternary RSL1D1/HDM2/p53 protein complex to promote p53 ubiquitination in CRC cells. The ensuing inactivation of p53 target genes, such as p21 and PUMA, attenuates cell cycle arrest and apoptosis, thereby promoting cell proliferation and survival (Fig. 9). On one hand, RSL1D1 directly downregulates the protein level of p53 by recruiting it to HDM2 for ubiquitination (Figs. 7 and 9). On the other hand, RSL1D1 indirectly downregulates the protein level of p53 by stabilizing HDM2 mRNA (Figs. 2, 4, 9, and Supplementary Fig. S3). The contrary regulatory mechanism is probably attributed to the different subnuclear localization of RSL1D1 in different types of cancer cells. Unlike in some cancer cell types [20], RSL1D1 is not confined to the nucleolus but distributes throughout the entire nucleus in CRC cells under normal conditions (Fig. 5), in which RSL1D1 performs more non-nucleolar functions, such as promoting tumor progression by negative regulation of p53 in this study.

Our results suggest that RSL1D1 is involved in regulating the mRNA stability of HDM2. As a nucleolar protein containing the ribosomal L1p/L10e domain in the N-terminus, RSL1D1 reportedly plays important ribosome-associated functions and participates in the regulation of the mRNA stability of NOLC1 and PTEN [19, 21]. Similarly, we found that RSL1D1 interacted with and stabilized HDM2 mRNA (Fig. 4), thereby leading to relatively high levels of HDM2 mRNA and protein in HCT116 cells in a p53-independent manner (Fig. 2).

Beyond ribosome-associated functions, our results also suggest a crucial non-ribosomal function of RSL1D1 as a novel p53-interacting protein. In this study, our data demonstrate that the p53-DBD directly interacts with both RSL1D1-CT and RSL1D1-NT and the RSL1D1-CT displays a stronger binding capability than the RSL1D1-NT (Fig. 6). The RSL1D1-NT also reportedly interacts with the RING finger domain in the C-terminus of HDM2 (aa 349–489) [20], whereas the N-terminus of HDM2 (aa 1–125) interacts with the 15–29 residues of p53 [62]. The complicated regulation and interaction between RSL1D1, p53, and HDM2 has significant biological importance. In HCT116^*p53+/+*^ cells, RSL1D1 recruits p53 to HDM2 via direct interaction to form a transient ternary protein complex (Fig. 7). The colocalization of RSL1D1, p53, and HDM2 enhances HDM2-mediated p53 ubiquitination, leading to a low level of p53 in CRC cells.

Our results also suggest a novel function of nucleolar proteins in the regulation of HDM2-mediated p53 ubiquitination and degradation. In the current opinion, nucleolar proteins play an important role in stabilizing p53. These proteins mainly include nucleolin [65], nucleostemin [66], NPM [67], ARF [68], and several protein components of large and small ribosomal subunits, such as RPL5 [69], RPL6 [11], RPL11 [70], RPL23 [71], RPL26 [72], RPS7 [73], and RPS14 [74]. In general, these nucleolar proteins are localized in the nucleolus under normal conditions. Upon nucleolar stress, they can be inducibly released to the nucleoplasm, where they interact with HDM2 and block HDM2-mediated p53 ubiquitination. Conversely, the findings in the current study demonstrate that RSL1D1 functions as an oncoprotein rather than a typical nucleolar protein in CRC cells. It is not only localized in the nucleolus but also in the entire nucleus under normal conditions (Fig. 5). This allows RSL1D1 to colocalize with p53 and HDM2 in a nucleolar stress-independent manner, which facilitates the formation of the ternary RSL1D1/HDM2/p53 complex, enhances HDM2-mediated p53 ubiquitination, and thereby regulates p53 negatively rather than positively (Figs. 5 and 7). In addition, our findings also provide evidences linking the highly expressed RSL1D1 (Supplementary Fig. S1) and p53 inactivation [75] in CRC cases.

Our results help explain the relatively low level of p53 protein in *p53+/+* human CRC cells [76, 77]. Generally, RSL1D1 normally maintains a relatively high expression level (Supplementary Fig. S1) and distributes throughout the nucleus (Fig. 5) in CRC cells. It negatively regulates p53 at the post-translational level by augmenting the expression and function of HDM2 (Figs. 2 and 3). As a result, a high level of RSL1D1 protein leads to a very low level of p53 protein in *p53+/+* CRC cells, which facilitates cell proliferation and survival (Fig. 1C). When the enhanced HDM2 function is inhibited by downregulating RSL1D1 or introducing HDM2- or p53-binding domains of RSL1D1 into cells to destroy the ternary protein complex, p53 ubiquitination decreases greatly (Fig. 3A and 7F). The decreased ubiquitination results in an increased amount of p53 protein (Fig. 2B and 7E), which induces G_1_ arrest and apoptosis (Fig. 1D and E).

Our results also suggest a potential target for drug development against colorectal neoplasms retaining wild-type p53. The tumor suppressor p53 is important in preventing cancer development [78]. HDM2, as a primary cellular inhibitor of p53 [53, 62, 79], binds and ubiquitinates p53 protein for nuclear export and proteasomal degradation [34, 80], thereby inhibiting p53 activity. Therefore, an important antitumor therapeutic strategy is to block the HDM2-p53 interaction to increase the amount of p53 protein. Over the past nearly two decades, scientists have made intense efforts to design and develop a number of structurally distinct, non-peptide, and highly potent small-molecule inhibitors of the HDM2-p53 protein-protein interaction or the HDM2 inhibitors [79], such as Idasanutlin [81], Nutlin-3a [82], RG7112 [83], MI-77301 [84], MI-888 [85], AMG-232 [86], RG7388 [87], NVP-CGM097 [88], and MK-8242 [89]. In the current study, RSL1D1 binds to HDM2 and p53 and upregulates HDM2 in CRC cells, implicating RSL1D1 as a potential antitumor target. In mouse xenograft models, siRSL1D1 treatment produced an excellent therapeutic efficacy in suppressing the growth of HCT116^*p53+/+*^ tumors (Fig. 8), demonstrating that downregulation of RSL1D1 is a highly efficient therapeutic strategy for treating CRC. In addition to gene silencing, blocking p53- or HDM2-RSL1D1 interaction is another potential treatment strategy for treating *p53+/+* colorectal tumors, since overexpression of the RSL1D1-NT or the RSL1D1-CT prevents p53 ubiquitination by competitively inhibiting the formation of the RSL1D1/HDM2/p53 protein complex (Fig. 7). Therefore, an important research avenue is to screen chemicals or biological RSL1D1 inhibitors that downregulate RSL1D1 or inhibit the p53- or HDM2-RSL1D1 protein-protein interaction, which may lead to an alternative to HDM2-targeted drugs.

## Conclusion

RSL1D1 distributed throughout the entire nucleus of CRC cells and negatively regulates nuclear p53. Crucially, RSL1D1 stabilizes HDM2 mRNA through protein-RNA interaction and also interacts with and recruits p53 to HDM2 to form a RSL1D1/HDM2/p53 protein complex, which enhances p53 ubiquitination and ultimately promotes the proliferation and survival of CRC cells. Both downregulation of RSL1D1 and destruction of the RSL1D1/HDM2/p53 complex can remarkably increase the cellular amount of p53 protein. Furthermore, RSL1D1 downregulation induces G1/S arrest and apoptosis in a p53-dependent manner by upregulating p21 and PUMA, thus reducing the growth of *p53+/+* CRC cells in vitro and in vivo. Our findings demonstrate that RSL1D1 is an oncoprotein in CRC and a potential molecular target for anticancer drug development.

## Supplementary Information

**Additional file 1: Supplementary Table S1.** Oligonucleotides in the current study.

**Additional file 2: Supplementary Fig. S1.** RSL1D1 Is Overexpressed in Human Colorectal Cancer. Data were obtained from the Oncomine Cancer Microarray database. Fold induction of RSL1D1 was set as 1.5 in the database interrogation of a variety of tumors versus normal controls (*P* < 0.001). Total number of datasets for analysis is listed below the heatmap. OE, overexpression; UE, underexpression.

**Additional file 3: Supplementary Fig. S2.** Downregulation of RSL1D1 Promotes the Expression of PUMA by Upregulation of FOXO3a in HCT116^*p53−/−*^ Cells. **A** The levels of RSL1D1 and FOXO3a proteins were evaluated by western blot analysis in HCT116^*p53+/+*^ and HCT116^*p53−/−*^ cells transfected with siRSL1D1 or siNC. β-actin was set as a loading control. **B** The levels of RSL1D1, FOXO3a, and PUMA proteins were evaluated in HCT116^*p53−/−*^ cells transfected with siNC+siNC, siRSL1D1 + siNC, or siRSL1D1 + siFOXO3a. β-actin was used as a loading control.

**Additional file 4: Supplementary Fig. S3.** RSL1D1 Regulates the HDM2-p53 Signaling Axis in HCT-8 Colorectal Cancer Cells. Cells were transfected with siRNA and harvested 48 h post-transfection. **A** The mRNA levels of RSL1D1, p53, HDM2, p21, and PUMA were determined by qRT-PCR in siRSL1D1- or siNC-transfected cells. GAPDH was used as an internal control to normalize the values. The normalized values of siNC-treated cells were set to 1. Data are represented as mean ± SD. Student’s t test. *P < 0.05 and **P < 0.01 denote significant difference. **B** The levels of RSL1D1, p53, HDM2, p21, and PUMA proteins were evaluated by western blot analysis in siRSL1D1- or siNC-transfected cells. β-actin was set as a loading control.

**Additional file 5: Supplementary Fig. S4.** Downregulation of RSL1D1 Does not Affect the Levels of HDM4 Protein in HCT116 Cells. The levels of RSL1D1 and HDM4 protein were determined by western blot analysis in HCT116^*p53+/+*^ and HCT116^*p53−/−*^ cells transfected with siRSL1D1 or siNC. β-actin was used as a loading control.

**Additional file 6: Supplementary Fig. S5.** Downregulation of HDM2 Does Not Remarkably Change the Expression of RSL1D1 in HCT116^*p53+/+*^ Cells. Cells were transfected with siRNA and harvested for determining the mRNA and protein levels of indicated genes 48 h post-transfection. **A** The mRNA levels of HDM2, RSL1D1, p53, p21, and PUMA were determined by qRT-PCR in siHDM2- or siNC-transfected cells. GAPDH was used as an internal control to normalize the values. The normalized values of siNC-treated cells were set to 1. Data are represented as mean ± SD. Student’s t test. *P < 0.05 and **P < 0.01 denote significant difference. **B** The levels of HDM2, RSL1D1, p53, p21, and PUMA proteins were evaluated by western blot analysis in siHDM2- or siNC-transfected cells. β-actin was set as a loading control.

**Additional file 7: Supplementary Fig. S6** Homemade Antibody against RSL1D1 Is Suitable for Immunofluorescence Assay. **A**, **B** IF assay was performed to detect RSL1D1 (red) in siRSL1D1-transfected (**A**) or RSL1D1-overexpressed (**B**) HCT116^*p53+/+*^ cells. The cells transfected with siNC (**A**) or overexpressing EGFP (**B**) were used as a negative control. Homemade anti-RSL1D1 monoclonal antibody was used as the primary antibody. The nuclei were stained with Hoechst (blue). Scale bars: 5 μm.

**Additional file 8: Supplementary Fig. S7.** Recombinant Proteins Were Purified by Affinity Chromatography and Subjected to SDS-PAGE Analysis to Assess the Purity. **A** Nucleotide sequences encoding full-length p53 and its truncated variants (aa 1–363, aa 1–292, aa 1–92, aa 293–393, and aa 93–292) were cloned into a prokaryotic expression vector pET-32a(+), respectively. Recombinant plasmids were transformed into *E. coli* BL21(DE3) and recombinant proteins were purified by affinity chromatography. The purified His-tagged proteins were subjected to SDS-PAGE analysis. **B** Nucleotide sequences encoding full-length RSL1D1 and its truncated variants (aa 1–281 and aa 282–485) were cloned into a prokaryotic expression vector pGEX-6P-1. Recombinant plasmids were transformed into *E. coli* BL21(DE3) and recombinant proteins were purified by affinity chromatography. The purified GST-tagged proteins were subjected to SDS-PAGE analysis. **C** Nucleotide sequence encoding HDM2 was cloned into a prokaryotic expression vector pET-32a-SUMO. Recombinant plasmids were transformed into *E. coli* BL21(DE3) and recombinant proteins were purified by affinity chromatography. The purified His-tagged protein was subjected to SDS-PAGE analysis.

## Data Availability

All data generated or analyzed during this study are included in this published article [and its supplementary information files].

## Abbreviations

5′-UTR: 5′-untranslated region
aa: Amino acid
Act-D: Actinomycin D
ARF: ADP-ribosylation factor
BAX: Bcl2-associated X protein
BiFC: Bimolecular fluorescence complementation
BSA: Bovine serum albumin
CRC: Colorectal cancer
CSIG: Cellular senescence-inhibited gene
CT: C-terminus
DBD: DNA binding domain
DMEM: Dulbecco’s modified Eagle’s medium
DMSO: Dimethylsulfoxide
DTT: Dithiothreitol
EGFP: Enhanced green fluorescent protein
FACS: Fluorescence-activated cell sorting
FITC: Fluorescein isothiocyanate
FL: Full-length
FOXO3a: Forkhead box O3A
GST: Glutathione-S-transferase
HDAC1: Histone deacetylase 1
HDM2: Human double minute 2
H&E: Hematoxylin and eosin
HRP: Horseradish peroxidase
IF: Immunofluorescence
IFA: Immunofluorescence assay
IP: Immunoprecipitation
MTT: 3-(4,5-dimethylthiazol-2-yl)-2,5-diphenyltetrazolium bromide
NOLC1: Nucleolar and coiled-body phosphoprotein 1
NPM: Nucleophosmin
NT: N-terminus
OD: Optical density
PBST: 1× PBS containing 0.1% Tween 20
PEI: Polyethylenimine
PI: Propidium iodide
PTEN: Phosphatase and tensin homolog
PUMA: p53 upregulated modulator of apoptosis
qRT-PCR: Quantitative real-time polymerase chain reaction
RIP: RNA immunoprecipitation
RP: Ribosomal protein
rRNAs: Ribosomal RNAs
RSL1D1: Ribosomal L1 domain-containing protein 1
SD: Standard deviation
SDS-PAGE: Sodium dodecyl sulfate polyacrylamide gel electrophoresis
siFOXO3a: siRNA against FOXO3a
siHDM2: siRNA against HDM2
siNC: Negative control siRNA
siRNA: Small interfering RNA
siRSL1D1: siRNA against RSL1D1
TIF-IA: Transcription initiation factor IA
UV: Ultraviolet
