# Supplementary material for: Ribosomal L1 domain-containing protein 1 coordinates with HDM2 to negatively regulate p53 in human colorectal Cancer cells
Source: J Exp Clin Cancer Res. 2021 Aug 6;40:245. doi: 10.1186/s13046-021-02057-8 (PMC8344204; doi:10.1186/s13046-021-02057-8)
Supplement: Supplementary file 1 — Additional file 1: Supplementary Table S1. Oligonucleotides in the current study. [file 13046_2021_2057_MOESM1_ESM.docx]

**Supplementary Table S1.** Oligonucleotides in the current study.

| Primers for qRT-PCR (human) | Sequences (5’-3’) |
| --- | --- |
| GAPDH F | TGGGCTACACTGAGCACCAG |
| GAPDH R | GGGTGTCGCTGTTGAAGTCA |
| RSL1D1 F | AGCCAAGCTCCGCCTTCT |
| RSL1D1 R | CCGCCTAATTCTGGCATCAG |
| p53 F | CCAGAAAACCTACCAGGGCA |
| p53 R | GAATGCAAGAAGCCCAGACG |
| HDM2 F | CCGGATCTTGATGCTGGTGT |
| HDM2 R | CTGATCCAACCAATCACCTGAAT |
| p21 F | AGCGATGGAACTTCGACTTTG |
| p21 R | CGAAGTCACCCTCCAGTGGT |
| PUMA F | GTCCTGTACAATCTCATCATGGGA |
| PUMA R | TCTGTGGCCCCTGGGTAAG |
| siRNA | Sequences (5’-3’) |
| siNC Sense | UUCUCCGAACGUGUCACGUTT |
| siNC Antisense | ACGUGACACGUUCGGAGAATT |
| siRSL1D1 Sense | CGAAGGAUGAACCCAAUUCAATT |
| siRSL1D1 Antisense | UUGAAUUGGGUUCAUCCUUCGTT |
| siHDM2 Sense | CUCUCGACUCAGAAGAUUAUATT |
| siHDM2 Antisense | UAUAAUCUUCUGAGUCGAGAGTT |
| siFOXO3a Sense | CAUGUUCAAUGGGAGCUUGGATT |
| siFOXO3a Antisense | UCCAAGCUCCCAUUGAACAUGTT |
