# Supplementary figures and images for: Ribosomal L1 domain-containing protein 1 coordinates with HDM2 to negatively regulate p53 in human colorectal Cancer cells
Source: J Exp Clin Cancer Res. 2021 Aug 6;40:245. doi: 10.1186/s13046-021-02057-8 (PMC8344204; doi:10.1186/s13046-021-02057-8)

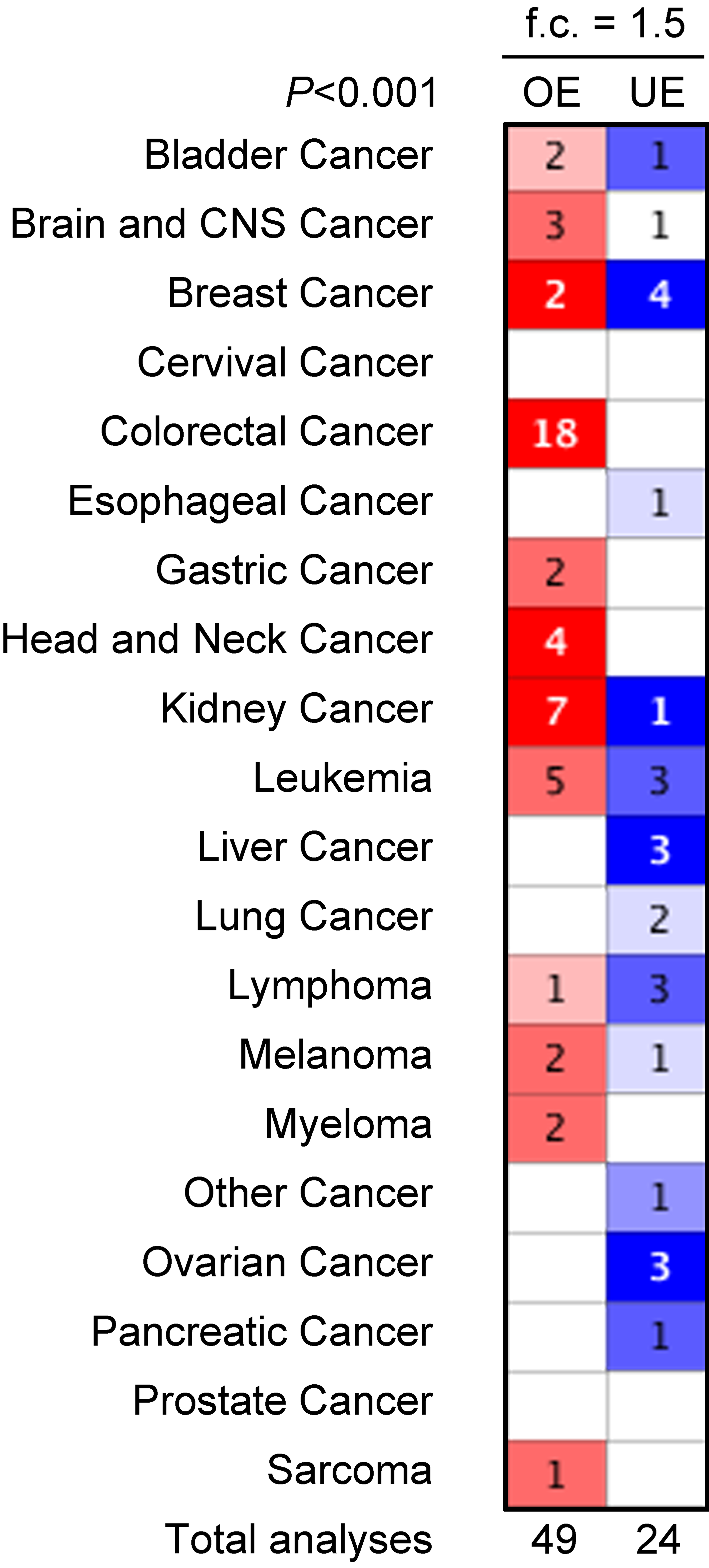

Supplement: Supplementary file 2 — Additional file 2: Supplementary Fig. S1. RSL1D1 Is Overexpressed in Human Colorectal Cancer. Data were obtained from the Oncomine Cancer Microarray database. Fold induction of RSL1D1 was set as 1.5 in the database interrogation of a variety of tumors versus normal controls (P < 0.001). Total number of datasets for analysis is listed below the heatmap. OE, overexpression; UE, underexpression. [file 13046_2021_2057_MOESM2_ESM.tif]

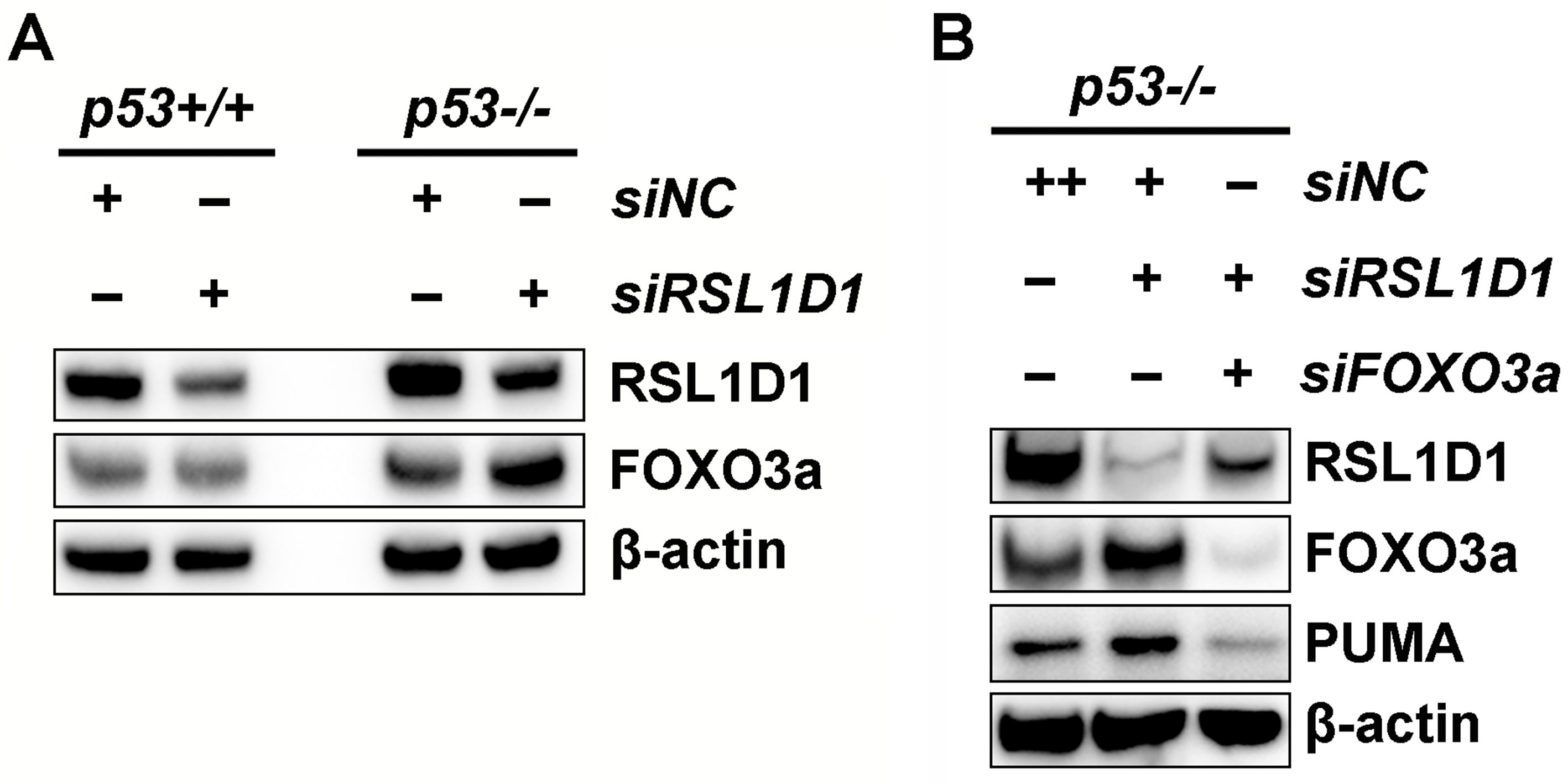

Supplement: Supplementary file 3 — Additional file 3: Supplementary Fig. S2. Downregulation of RSL1D1 Promotes the Expression of PUMA by Upregulation of FOXO3a in HCT116p53−/− Cells. A The levels of RSL1D1 and FOXO3a proteins were evaluated by western blot analysis in HCT116p53+/+ and HCT116p53−/− cells transfected with siRSL1D1 or siNC. β-actin was set as a loading control. B The levels of RSL1D1, FOXO3a, and PUMA proteins were evaluated in HCT116p53−/− cells transfected with siNC+siNC, siRSL1D1 + siNC, or siRSL1D1 + siFOXO3a. β-actin was used as a loading control. [file 13046_2021_2057_MOESM3_ESM.tif]

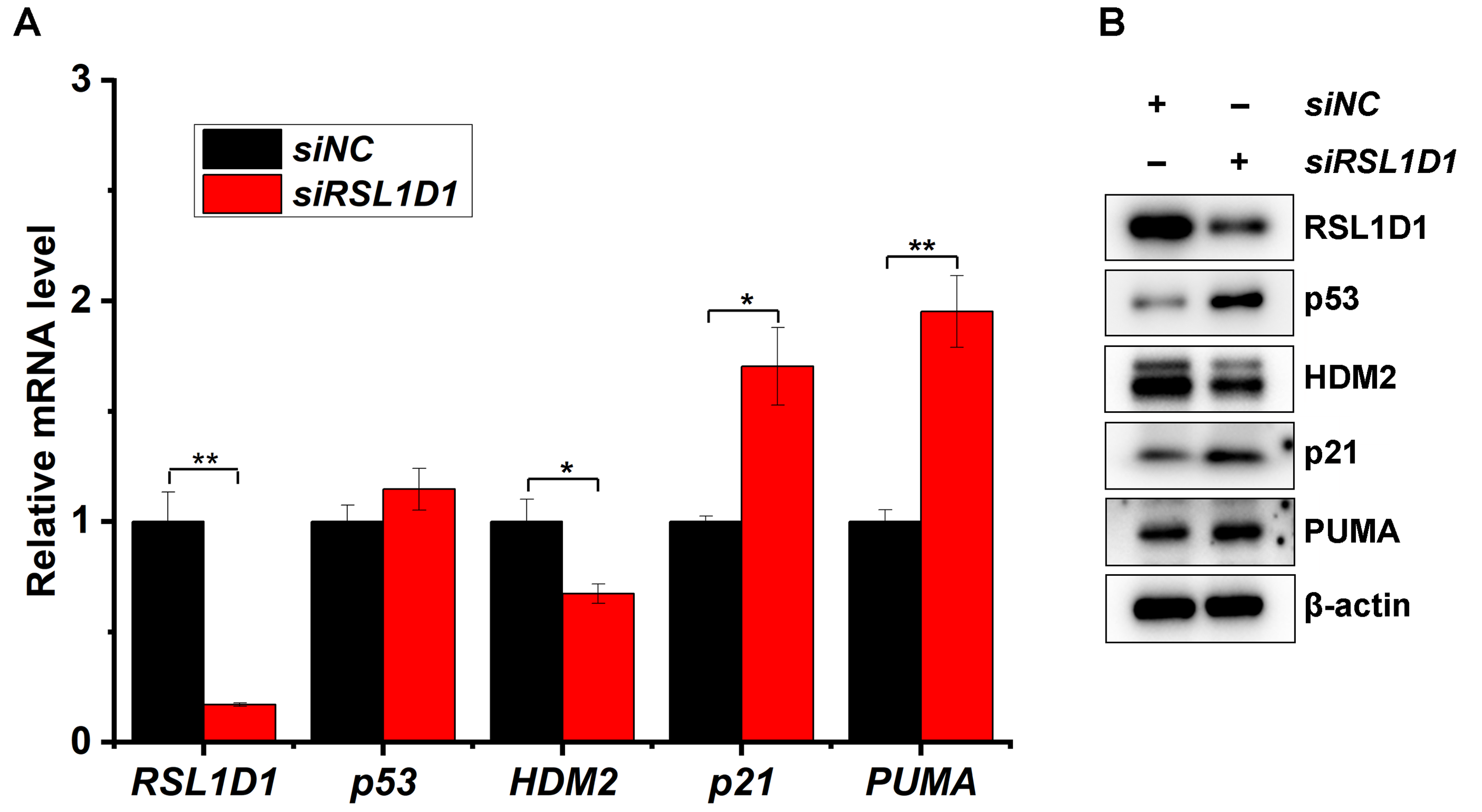

Supplement: Supplementary file 4 — Additional file 4: Supplementary Fig. S3. RSL1D1 Regulates the HDM2-p53 Signaling Axis in HCT-8 Colorectal Cancer Cells. Cells were transfected with siRNA and harvested 48 h post-transfection. A The mRNA levels of RSL1D1, p53, HDM2, p21, and PUMA were determined by qRT-PCR in siRSL1D1- or siNC-transfected cells. GAPDH was used as an internal control to normalize the values. The normalized values of siNC-treated cells were set to 1. Data are represented as mean ± SD. Student’s t test. *P < 0.05 and **P < 0.01 denote significant difference. B The levels of RSL1D1, p53, HDM2, p21, and PUMA proteins were evaluated by western blot analysis in siRSL1D1- or siNC-transfected cells. β-actin was set as a loading control. [file 13046_2021_2057_MOESM4_ESM.tif]

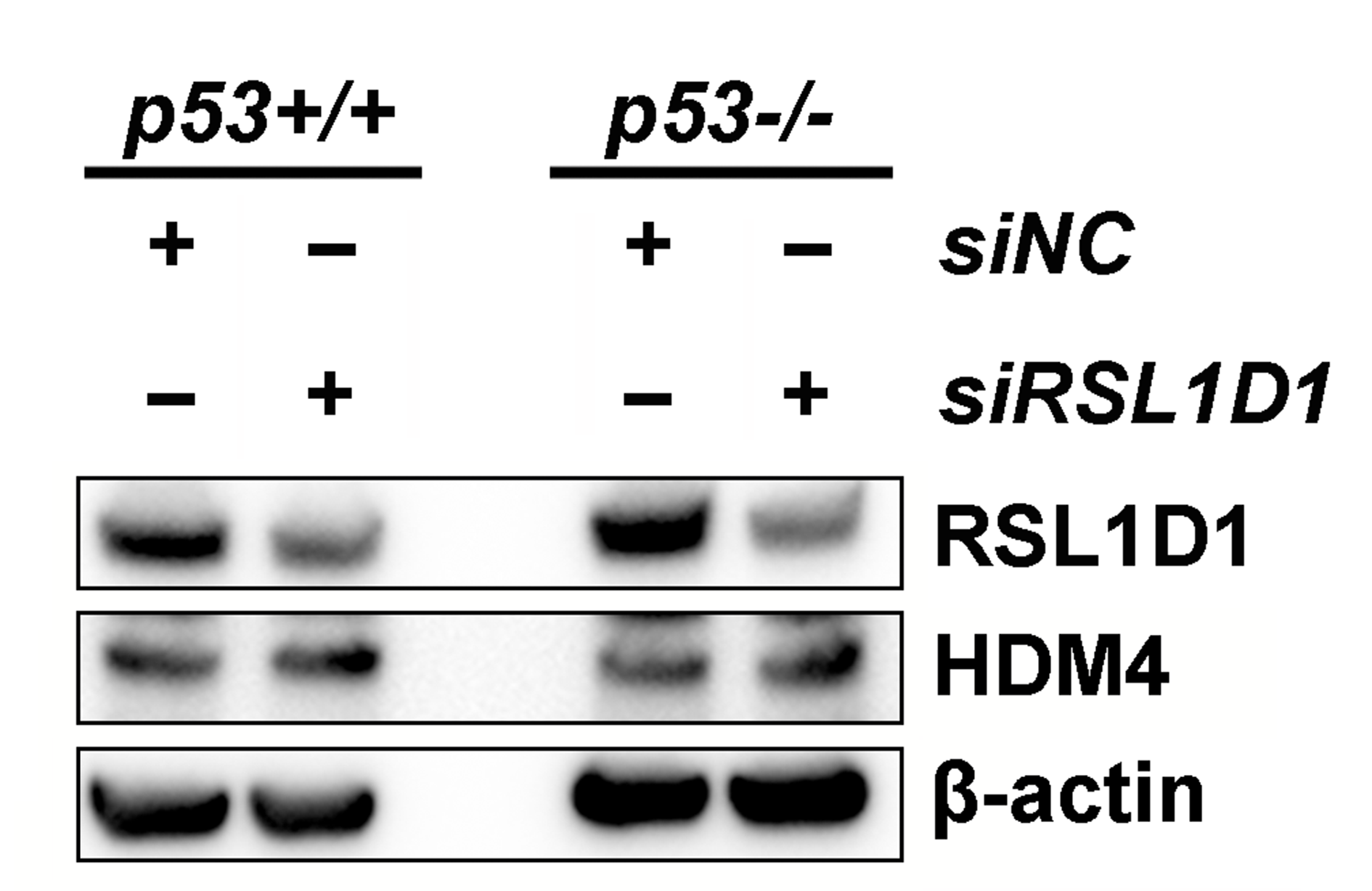

Supplement: Supplementary file 5 — Additional file 5: Supplementary Fig. S4. Downregulation of RSL1D1 Does not Affect the Levels of HDM4 Protein in HCT116 Cells. The levels of RSL1D1 and HDM4 protein were determined by western blot analysis in HCT116p53+/+ and HCT116p53−/− cells transfected with siRSL1D1 or siNC. β-actin was used as a loading control. [file 13046_2021_2057_MOESM5_ESM.tif]

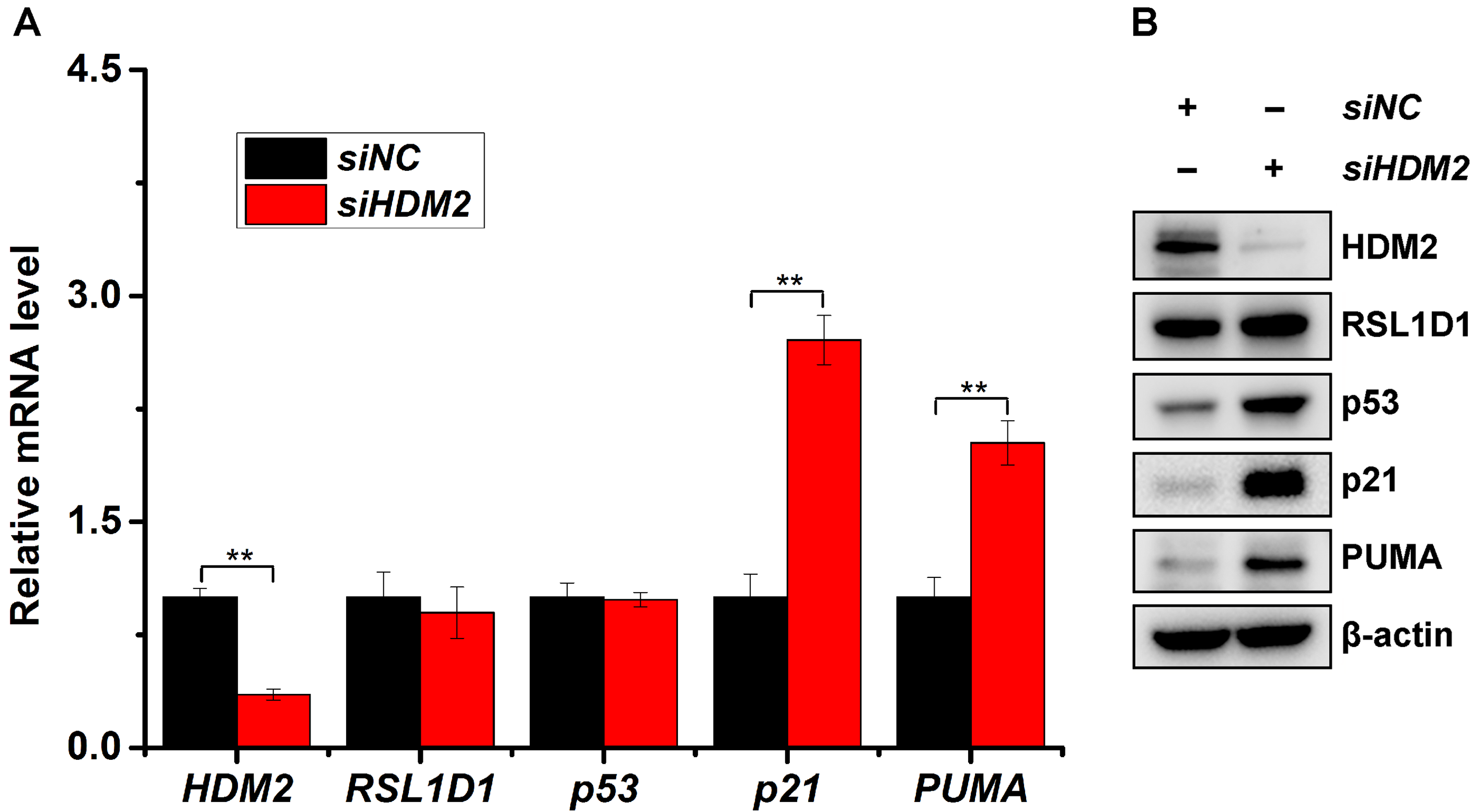

Supplement: Supplementary file 6 — Additional file 6: Supplementary Fig. S5. Downregulation of HDM2 Does Not Remarkably Change the Expression of RSL1D1 in HCT116p53+/+ Cells. Cells were transfected with siRNA and harvested for determining the mRNA and protein levels of indicated genes 48 h post-transfection. A The mRNA levels of HDM2, RSL1D1, p53, p21, and PUMA were determined by qRT-PCR in siHDM2- or siNC-transfected cells. GAPDH was used as an internal control to normalize the values. The normalized values of siNC-treated cells were set to 1. Data are represented as mean ± SD. Student’s t test. *P < 0.05 and **P < 0.01 denote significant difference. B The levels of HDM2, RSL1D1, p53, p21, and PUMA proteins were evaluated by western blot analysis in siHDM2- or siNC-transfected cells. β-actin was set as a loading control. [file 13046_2021_2057_MOESM6_ESM.tif]

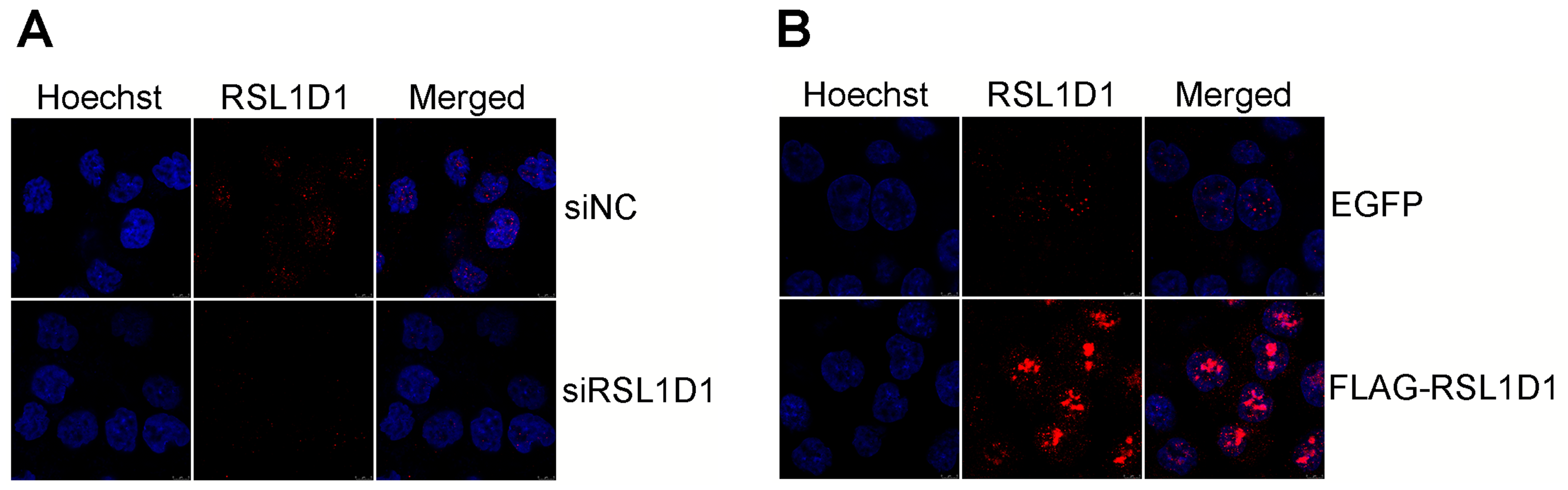

Supplement: Supplementary file 7 — Additional file 7: Supplementary Fig. S6 Homemade Antibody against RSL1D1 Is Suitable for Immunofluorescence Assay. A, B IF assay was performed to detect RSL1D1 (red) in siRSL1D1-transfected (A) or RSL1D1-overexpressed (B) HCT116p53+/+ cells. The cells transfected with siNC (A) or overexpressing EGFP (B) were used as a negative control. Homemade anti-RSL1D1 monoclonal antibody was used as the primary antibody. The nuclei were stained with Hoechst (blue). Scale bars: 5 μm. [file 13046_2021_2057_MOESM7_ESM.tif]

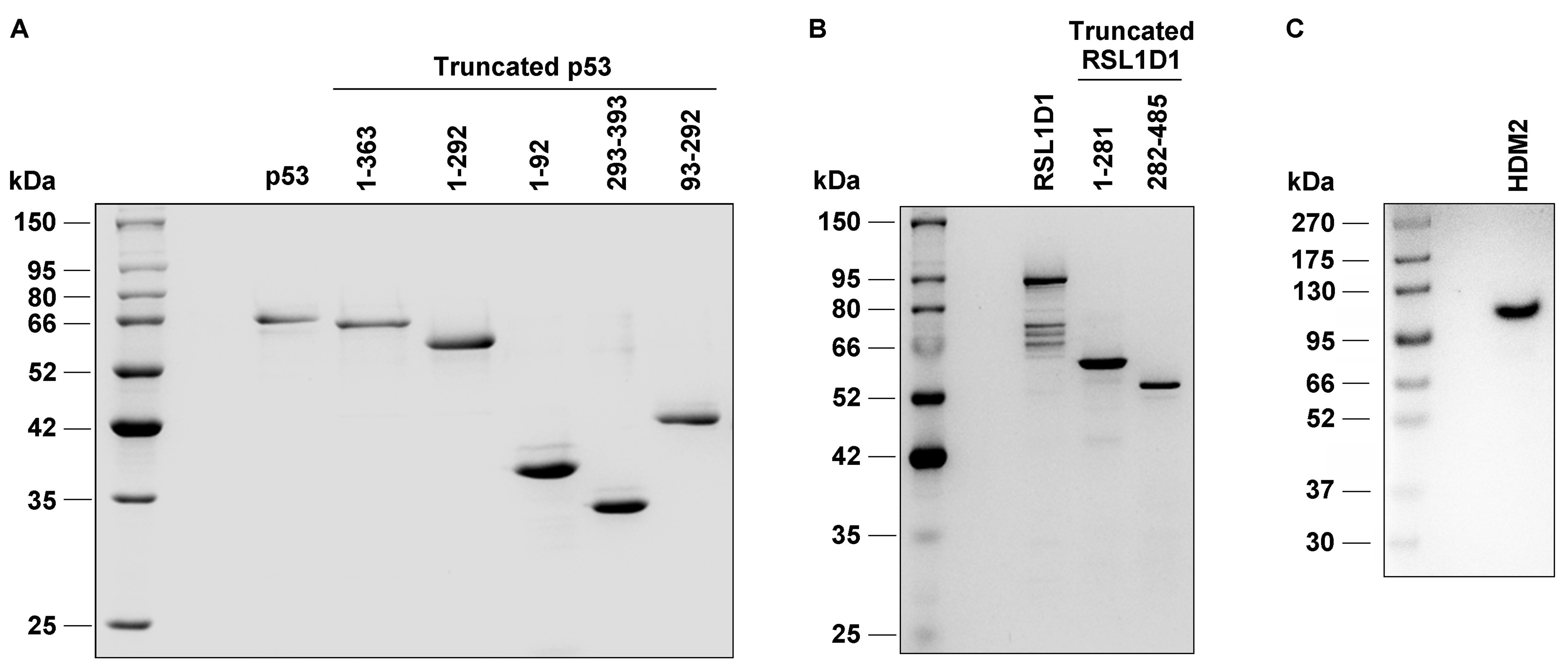

Supplement: Supplementary file 8 — Additional file 8: Supplementary Fig. S7. Recombinant Proteins Were Purified by Affinity Chromatography and Subjected to SDS-PAGE Analysis to Assess the Purity. A Nucleotide sequences encoding full-length p53 and its truncated variants (aa 1–363, aa 1–292, aa 1–92, aa 293–393, and aa 93–292) were cloned into a prokaryotic expression vector pET-32a(+), respectively. Recombinant plasmids were transformed into E. coli BL21(DE3) and recombinant proteins were purified by affinity chromatography. The purified His-tagged proteins were subjected to SDS-PAGE analysis. B Nucleotide sequences encoding full-length RSL1D1 and its truncated variants (aa 1–281 and aa 282–485) were cloned into a prokaryotic expression vector pGEX-6P-1. Recombinant plasmids were transformed into E. coli BL21(DE3) and recombinant proteins were purified by affinity chromatography. The purified GST-tagged proteins were subjected to SDS-PAGE analysis. C Nucleotide sequence encoding HDM2 was cloned into a prokaryotic expression vector pET-32a-SUMO. Recombinant plasmids were transformed into E. coli BL21(DE3) and recombinant proteins were purified by affinity chromatography. The purified His-tagged protein was subjected to SDS-PAGE analysis. [file 13046_2021_2057_MOESM8_ESM.tif]
